# The role of Transposable Elements in shaping the combinatorial interaction of Transcription Factors

**DOI:** 10.1186/1471-2164-13-400

**Published:** 2012-08-16

**Authors:** Alessandro Testori, Livia Caizzi, Santina Cutrupi, Olivier Friard, Michele De Bortoli, Davide Cora', Michele Caselle

**Affiliations:** 1 Center for Molecular Systems Biology, University of Turin, c/o IRCC - Str. Prov. 142 Km. 3.95, Turin, Candiolo, I-10060, Italy; 2 Bioindustry Park Silvano Fumero, Colleretto Giacosa, Italy; 3 Department Oncological Sciences, University of Turin, Str. Prov. 142 Km. 3.95, Turin, I-10060, Italy; 4 Systems Biology Lab, Institute for Cancer Research and Treatment (IRCC), Str. Prov. 142 Km. 3.95, Turin, Candiolo, I-10060, Italy; 5 Department of Life Sciences and Systems Biology, University of Turin, v. Acc. Albertina 13, Turin, 10123, Italy; 6 Department of Physics, University of Turin, v. P. Giuria 1, Turin, I-10125, Italy

**Keywords:** Transposable elements, ChIP-seq, Transcription factors, ERα, Combinatorial interaction

## Abstract

**Background:**

In the last few years several studies have shown that Transposable Elements (TEs) in the human genome are significantly associated with Transcription Factor Binding Sites (TFBSs) and that in several cases their expansion within the genome led to a substantial rewiring of the regulatory network. Another important feature of the regulatory network which has been thoroughly studied is the combinatorial organization of transcriptional regulation. In this paper we combine these two observations and suggest that TEs, besides rewiring the network, also played a central role in the evolution of particular patterns of combinatorial gene regulation.

**Results:**

To address this issue we searched for TEs overlapping Estrogen Receptor α (ERα) binding peaks in two publicly available ChIP-seq datasets from the MCF7 cell line corresponding to different modalities of exposure to estrogen. We found a remarkable enrichment of a few specific classes of Transposons. Among these a prominent role was played by MIR (Mammalian Interspersed Repeats) transposons. These TEs underwent a dramatic expansion at the beginning of the mammalian radiation and then stabilized. We conjecture that the special affinity of ERα for the MIR class of TEs could be at the origin of the important role assumed by ERα in Mammalians. We then searched for TFBSs within the TEs overlapping ChIP-seq peaks. We found a strong enrichment of a few precise combinations of TFBS. In several cases the corresponding Transcription Factors (TFs) were known cofactors of ERα, thus supporting the idea of a co-regulatory role of TFBS within the same TE. Moreover, most of these correlations turned out to be strictly associated to specific classes of TEs thus suggesting the presence of a well-defined "transposon code" within the regulatory network.

**Conclusions:**

In this work we tried to shed light into the role of Transposable Elements (TEs) in shaping the regulatory network of higher eukaryotes. To test this idea we focused on a particular transcription factor: the Estrogen Receptor α (ERα) and we found that ERα preferentially targets a well defined set of TEs and that these TEs host combinations of transcriptional regulators involving several of known co-regulators of ERα. Moreover, a significant number of these TEs turned out to be conserved between human and mouse and located in the vicinity (and thus candidate to be regulators) of important estrogen-related genes.

## Background

It is well known that Transcription Factors (TFs in the following) exert their regulatory function in a combinatorial and cooperative way [1]. This is particularly true for higher eukaryotes where simple and general arguments borrowed from information theory show that combinatorial interactions among TFs is a mandatory consequence of the lack of information of their binding sequences [2].

Thanks to the recent advances in ChIP-seq technologies and in particular to the so called Re-Chip experiments [3], it is now possible to address cooperative interactions among TFs in a quantitative way. An interesting open problem in this context is to understand the evolutionary mechanisms which led to clustering of binding sequences of the “right” TFs in the regulatory regions of target genes. While it is easy to create a single binding sequence by point mutation, the appearance of a combination of several binding motifs in an extended region of DNA is definitely more unlikely in this way. Indeed, it is difficult to understand how a *local* evolutionary process could create an extended (*non-local*) combination of binding sequences. A possible solution of this apparent paradox is to assume that a suitable template for the sought combination of motifs already exists in the genome and is then transferred as a whole in the regulatory region of the target genes.

Transposable Elements (TEs in the following) are the natural candidates to play this role [4-7].

In agreement with the above observation, in the last few years several ChIP-seq analyses have shown that TEs in the human genome are significantly enriched with transcription factor binding sites [8-13]. This was shown for various master TFs (p53, POU5F1, SOX2, C-Myc, OCT4, NANOG) and, among others, for ERα, which is the main focus of our paper. It is interesting to notice that this association with TEs persists even when a total affinity approach is used to reconstruct the transcriptional regulatory network [14].

This association between TFs and TEs suggests that transposition events could have played a central role in the emergence and success of combinatorial gene regulation in complex eukaryotes. According to this hypothesis, when a TE with a “good” combination of binding sequences is transposed near the regulatory region of a target gene it is “exapted” [15] and then conserved by evolution. Stochastic mutation of the original transposable sequence can then fine tune the regulatory module by eliminating or adding suitable binding motifs. Indeed there is clear evidence that several TE families are under strong purifying selection [16,17] and that most of the known CNEs (conserved non coding elements) are located inside TEs [18].

Bioinformatic tools can only identify the most recent exaptation events, for which the underlying TE can be recognized. An extreme version of this hypothesis would suggest that also the regulatory regions for which no TE structure is visible are actually the modern remnant of ancient exaptation events, in which the underlying TE structure was deleted by evolution.

A consequence of this picture is that, due to continuous transposition events, the regulatory network of higher Eukaryotes is characterized by an extensive and fast rewiring of regulatory interactions [8,12,19,20].

In this paper we test this picture relative to a specific case of inducible TF, that is the Estrogen Receptor α (ERα). Besides looking for TEs enrichments and transcription factor binding sites (TFBSs) correlations we also sought to understand if the binding pattern (and the preference for specific families of TEs) of ERα varies as a consequence of the kind of experimental activation of ERα.

ERα plays a central role in the development and function of mammary glands and other sex hormone-regulated districts and is a key molecular player in most human breast cancers. Indeed, antiestrogenic drugs are among the most widely and successfully used treatments since long time. In vitro stimulation of human breast cancer derived cell lines with 17β-estradiol (“E2T” condition in the following) evokes rapid and massive binding of ERα to the genome and regulation of transcription of thousands of genes [21-23] and consequently stimulates proliferation. However, these cell lines are made proliferating also by continuous culture in so-called “complete medium” (“CM” condition in the following) that contains low level estrogen derived from the 5 to 10% fetal calf serum added to this medium. These two modalities represent “chronic” (CM) exposure to low doses *versus* “acute” (E2T) exposure to elevated levels of hormones and may represent two extreme modalities for cells to respond to the hormone, which display both common and specific ERα binding sites. It should also be stressed that human reproductive biology is quite different from other Mammals in terms of cycling of hormones. Estrogen receptor binding sites profiling of breast cancers is associated with the clinical outcome under antiestrogen treatment, further suggesting that estrogen concentration may correlate with changes in binding sites [24]. For all these reasons ERα is a perfect candidate to test our proposals.

On these grounds, we present here a comparative analysis of ERα binding sequences in chronic *versus* acute stimulation, in order to seek for common or differential TE enrichment and TFBSs composition.

## Results

In order to test our proposals we analyzed two publicly available datasets corresponding to the two conditions (CM and E2T) described above [24,25]. Details on the datasets are available in the Methods section. We report here the results of a set of enrichment tests performed on these data. Details on the tests are also available in the Methods section and in the Additional file 1.

ERα preferentially binds DNA within particular classes of TE sequences in both CM and E2T conditions. While some of the selected TEs are enriched in both conditions, a few of them are specifically enriched only in CM or E2T datasets.

**Figure 1:**
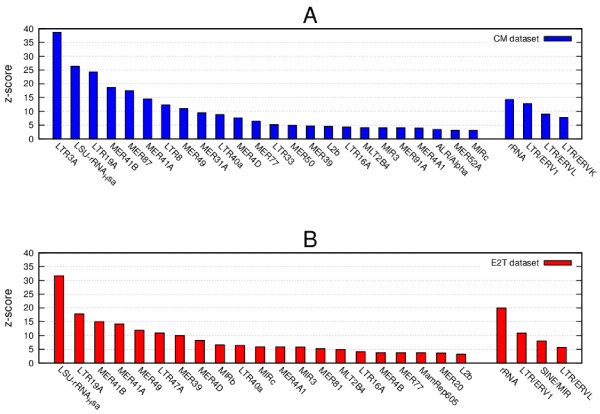
**Enrichment of TEs in the ERα binding datasets.** Histogram of the z-score values showing the enrichment, with respect to random reshuffling, of single TEs (left) and classes (right) in the CM dataset (**A**) and in the E2T dataset (**B**) respectively. Only values z > 3 are shown.

**Figure 2:**
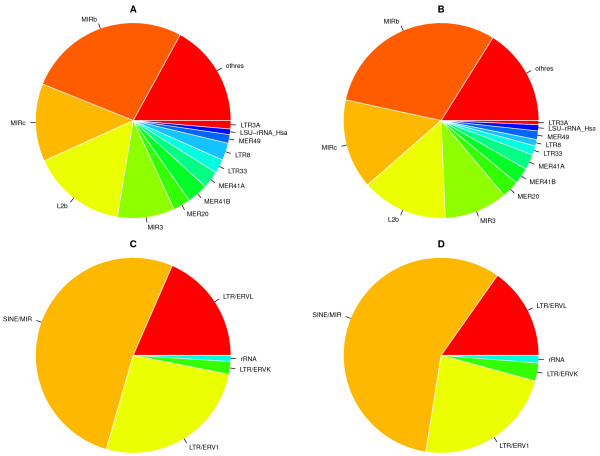
**Relative fractions of enriched single TEs and classes.** Pie-charts showing the relative fraction of enriched single TEs and classes shown in Figure 1A and Figure 1B. Top pies: single TEs; bottom pies: classes; left pies: CM dataset; right pies: E2T dataset.

We show the results of our enrichment test in Figure 1A for the “CM” dataset [24] and in Figure 1B for the “E2T” dataset [25] (see also Additional file 2: Table S3 and Additional Table S4 for absolute values and Figure 2 for distributions).

The very high levels of enrichment observed prove that ERα preferentially binds DNA within a few specific families of TEs both in CM and in E2T conditions. This confirms previous results for ERα reported in [9] (which were based on a different ChIP-seq dataset) and agrees with what already observed for p53 [9,10], POU5F1 also known as OCT4 [9,12], SOX2 [9], C-Myc [11], NANOG [12] and STAT1 [13].

Examining results in more detail, we see that the TEs are not all on the same ground and that the highest enrichment scores are reached for a few selected TE families. This suggests that besides a general affinity for the given TE class, ERα is able to perform a fine-tuned selection and shows a specific affinity for a few well-defined small families of TEs within the class. This remarkable selectivity allowed us to perform a detailed analysis of the combinatorial regulatory patterns involving ERα and to identify a few putative new co-regulators. Our results suggest that each particular TE family within a given class should actually correspond to a different combinatorial regulatory pattern.

As far as the difference between the two datasets is concerned, when comparing the enrichment scores we clearly see a twofold pattern. The ERV-like TEs (in particular those belonging to the ERV1 and ERVL classes) are enriched in both datasets while the MIR-like TEs show a preferential enrichment in the E2T dataset. In order to test this observation further we performed the same enrichment analysis separately for the three groups of sequences: i) intersection of the two datasets, ii) the binding events present only in the CM dataset and iii) the binding events present only in the E2T dataset. The results are reported in Table 1 and nicely confirm the previous results.

**Table 1:** z-scores (first five columns) and numbers of instances (last five columns) of the most enriched transposable elements and classes of TEs for the five possible combinations of the two datasets: CM, E2T, CM only (= CM\ E2T), E2T only (= E2T\ CM) and the intersection of the two datasets

	**CM**	**CM only**	**E2T**	**E2T only**	**int**	**CM**	**CM only**	**E2T**	**E2T only**	**int**
ALR/Alpha	3.39	4.56	-2.32	-2	-1.63	19	19	0	0	0
HAL1-2a_MD	-1.73	-0.9	2.06	4.61	-1.84	1	1	10	10	0
L1PBa	2.46	5.42	-2.33	-2.12	-1.24	15	13	2	0	2
L2b	4.54	5.01	3.18	2.76	1.02	284	149	292	157	135
LSU-rRNA_Hsa	26.31	11.67	31.65	16.3	21.52	17	5	21	9	12
LTR16A	4.31	1.17	4.1	1.51	4.39	30	9	32	11	21
LTR19A	24.31	17.77	17.83	8.8	17.7	22	10	17	5	12
LTR3	26.63	9.93	27.34	9.12	25.12	12	3	12	3	9
LTR33	5.14	3.2	2.04	-0.53	3.79	42	18	31	7	24
LTR3A	38.69	29.61	21.24	4.95	21.92	25	14	14	3	11
LTR40a	8.77	8.45	6.38	4.28	6.19	20	10	17	7	10
LTR47A	6.12	-0.1	10.88	6.51	8.63	10	2	17	9	8
LTR8	12.3	15.3	2.68	1.24	3.02	52	38	22	8	14
MER11A	1.87	6.17	-1.71	-0.89	-1.8	8	8	1	1	0
MER20	2.84	0.51	3.6	2.71	2.91	52	20	59	27	32
MER21A	3.21	4.28	0.95	1.78	0.5	13	9	9	5	4
MER31A	9.47	5.65	6.36	0.97	9.56	20	7	16	3	13
MER39	4.67	1.74	9.96	8.59	5.65	18	4	33	19	14
MER39B	5.28	0.93	7.14	4.31	6.69	11	2	15	6	9
MER41A	14.46	6.04	14.16	5.75	15.04	52	15	52	15	37
MER41B	18.63	8.54	14.97	5.84	16.22	63	23	56	16	40
MER49	10.95	1.98	11.85	2.15	15.15	26	3	29	6	23
MER4A	4.12	2.47	6.85	7.26	3.65	10	4	15	9	6
MER4A1	3.9	0.32	5.87	5.09	3.96	15	5	21	11	10
MER4B	-0.89	-1.52	3.75	5.06	0.12	6	1	21	16	5
MER4D	7.58	1.72	8.2	1.84	9.92	19	3	22	6	16
MER50	4.9	5.86	2.42	2.29	1.73	21	13	16	8	8
MER52A	3.07	5.93	-0.35	0.09	-0.61	16	13	7	4	3
MER77	6.43	5.66	3.74	1.84	3.7	22	12	17	7	10
MER81	-0.59	-1.26	5.19	5.96	0.81	5	0	21	16	5
MER87	17.46	9.3	16.67	7.78	13.82	15	6	14	5	9
MER91A	3.99	4.67	2.59	1.18	2.24	17	9	15	7	8
MIR3	4.01	-0.03	5.81	3.11	5.23	172	55	214	97	117
MIRb	2.76	1.19	6.57	5.45	3.43	488	190	625	327	298
MIRc	3.05	2.04	5.89	5.56	2.66	233	95	304	166	138
MLT2B1	2.31	3.06	0.84	-0.23	0.98	15	8	12	5	7
MLT2B4	4.02	1.67	4.87	2.45	3.99	21	7	25	11	14
MamRep605	1.97	0.59	3.73	2.61	2.24	13	4	19	10	9
Plat_L3	2.64	3.61	1.34	1.29	0.62	13	8	11	6	5
LTR/ERV1	12.77	5.84	10.86	4.04	11.55	592	227	604	239	365
LTR/ERVK	7.7	7.32	1.52	-0.95	3.15	77	45	47	15	32
LTR/ERVL	8.98	5.6	5.65	1.37	6.83	415	177	395	157	238
LTR/Gypsy?	3.01	2.49	2.55	0.5	2.76	24	9	24	9	15
SINE/MIR	2.56	0.97	8.02	7.33	3.02	1170	480	1486	796	690
Satellite/centr	3.51	5.22	-2.71	-2.19	-1.89	23	23	0	0	0
rRNA	14.31	9.74	19.99	17.64	12.42	23	9	33	19	14

In the first part of the table are reported TEs which are enriched (i.e. have a z-score greater than 3) in at least one of the two datasets used for the analysis. In the first five columns we report the z-score for the five possible combinations of the two datasets: CM, E2T, CM only = CM\ E2T, E2T only = E2T\ CM and the intersection of the two datasets. In the last five columns the absolute number of instances of each enriched TE in each one of the five combinations of the datasets. In the second part of the table we report the same information for the classes of TEs enriched in both the datasets used for the analysis.

We see two possible explanations for the different behaviour in the two datasets. They correspond to different experimental settings: the E2T dataset corresponds to an acute, sudden exposure to elevated concentration of 17β-estradiol, while the CM dataset corresponds to cells that are grown in a steady state situation of low estrogen concentration. Therefore, either i) the two different exposures to estrogen likely correspond to different concentrations of the Estrogen Receptor in the nucleus, thus making it possible that also binding sequences with a lower affinity to the receptor are bound in the dataset (E2T) with the highest concentration; or ii) continuous *versus* pulse estrogen exposure would result in different concentration and activity of cofactors known to play a crucial role in fine tuning the binding of ERα to its target sequences.

Most of the enriched classes of TEs predate the human-mouse separation but a few of them appeared in the last 100 Myrs and induced a rewiring of the human regulatory network with respect to the murine one.

An interesting feature of the above results is that most of the enriched TE families are rather old Transposons. This is particularly true for the MIR-like sequences, whose amplification is known to predate (at least in part) the mammalian radiation [26]. In the case of the ERV-like sequences the time window is slightly larger. TEs belonging to the ERVL class, which is the largest class of ERV sequences, and is enriched in both our datasets, ceased their activities only 40 Myrs ago [27], while TEs belonging to the ERVK class (which is enriched only in the CM dataset) are definitely younger. A tentative estimate of their mean age gives numbers ranging from 8 Myrs (if only human ERVK are considered) up to 18 Myrs (if human and chimp ERVK are considered) [28,29]. Even if it is widely accepted that these TEs are no longer active, examples of very recent ERVK insertions (about 6 Myrs old) exist [30]. In any case it is clear that most of the ERVK insertions occurred after the new world/old world monkeys split.

Thus it seems that, as far as these TEs binding sequences are concerned, a twofold picture exists also for the network rewiring.

A large portion of the ERα regulatory network (and in particular the part which is activated in the E2T condition) underwent a dramatic rewiring at the beginning of the mammalian radiation (more than 100 Myrs ago) but then it stabilized, and is now shared by all the Mammalians. This obviously does not exclude local changes and possibly also transposition events after the mouse/human divergence, but suggests that these should not be the rule and that a massive rewiring of this subnetwork in the last 100 Myrs should be excluded. Finding evidences of this conservation by a direct comparison of the human and mouse sequences is very difficult because most of these ancient TEs are too corrupted to be identified. Nonetheless, by combining the syntenic maps and the TE annotations of the human and mouse genomes downloaded from the Ensembl database [31] we were able to single out, among the TEs bound by ERα in our datasets, several pairs of conserved Trasposons (our results are summarized in Additional file 2: Tables S6 and S7). These examples are of particular interest since the very fact that they could be identified suggests that a large portion of the TE sequence should be under purifying selection and, therefore, they may represent important regulatory sequences. As expected from the above discussion, most of the conserved TEs belong to MIR-like classes (since most of the ERV type TEs are younger than the human-mouse separation) with a few notable exceptions like MER20, MER31A and MER77 which are known to be among the oldest ERV-type TEs.

Among the TEs enriched in our dataset belonging to the MIR family, a fraction between 15% and 25% of the TEs was conserved between human and mouse. We then looked for putative regulated genes of these conserved MIR-like TE’s (details in the Methods section). We found several putatively regulated genes (see Additional file 2: Table S10 and S11) and, remarkably enough, most of them turned out to be important known targets or cofactors of ERα. In particular, among the regulated genes, we found GREB1 (growth regulation by estrogen in breast cancer 1 gene) which is known to be regulated by ER and to be involved in estrogen-responsive breast cancer (see for instance [32]); RARα, which is a known cofactor of ERα [3]; TAP/SEC14L2 which is known to be downregulated by ER in breast cancer cells [33]; GLUT1/SLC2A1, whose expression in the luminal epithelial cells of the uterus was shown to be regulated by ERα [34] and is involved in the ERα mediated response to hypoxia [35] and several other ERα regulated genes like Anxa6 [36], Fyn [37], CA12 [38], CYP1B1 [39,40], KRT13 [41] and PRKACA [42].

These findings suggest that also some of the remaining genes targeted by conserved TEs could be involved in ERα regulated pathways and that this combination of ChIP-seq and evolutionary conserved TEs could be in general (i.e. also for the other TFs associated TEs dicussed in the introduction) a powerful tool to identify reliable targets.

On the contrary, the portion of the regulatory network that is activated in CM conditions, which is strongly enriched in ERVL and ERVK insertions, most probably underwent a significant rewiring in the last 100 Myrs. However, note that, as above, also in this case one cannot exclude local cases of convergent evolution (see below for a nice example) or that part of these insertions (being too young to have been modeled by evolution) simply correspond to non-functional bindings, thus mitigating the rewiring effect of the ERV-like transposons expansion.

### MIR-like and ERV-like TEs targeted by ERα are preferentially located near the TSS of regulated genes

**Figure 3:**
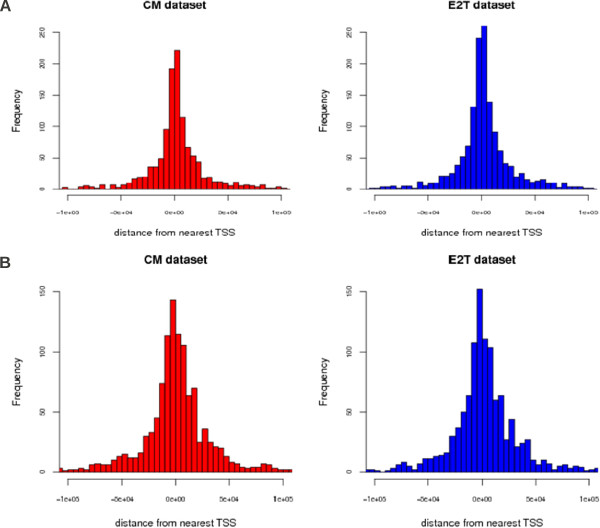
**Selected classes of TEs are located nearby the TSS of known genes.** (**A**) Histogram of the distance of MIR-like TEs targeted by ERα from the TSS of the nearest gene. (**B**) Histogram of the distance of ERV-like TEs targeted by ERα from the TSS of the nearest gene.

In order to support the idea that the MIR-like and ERV-like TEs in the peak datasets have a regulatory role, we identified their chromosomal localization and measured their distance from the Transcription Start Site (TSS) of the nearest gene. Results of this analysis are reported in Figure 3A for MIR-like TEs and in Figure 3B for ERV-like TEs. We see that in both cases the TEs nicely peak around the TSS. The pattern is very similar in the two datasets and supports our suggestion that these TEs were indeed exapted by the Estrogen Receptor and are likely to play a regulatory role in driving the response to estrogen stimulation.

### TEs selected by ERα are enriched for binding sequences of TFs representing known cofactors of ERα. Our approach allows the identification of new putative cofactors

In order to exert its regulatory functions, ERα very often interacts with other TFs. In particular, clear evidences of regulatory interactions with FoxA1, RARα, GATA3, SP1 and AP1 (JUN + FOS) have been recently observed ( [3,25,43-47]).

In order to test whether these interactions are mediated by TEs and in particular by those that are enriched in the two datasets, we evaluated the Transposon Affinity Score (TAS) value (for the definition see the Methods section) for each TF. Results are reported in Additional file 2: Table S3 and S4 separately for the two datasets (only TFs with TAS value greater than 0.1 for at least one of the enriched TEs are considered).

**Figure 4:**
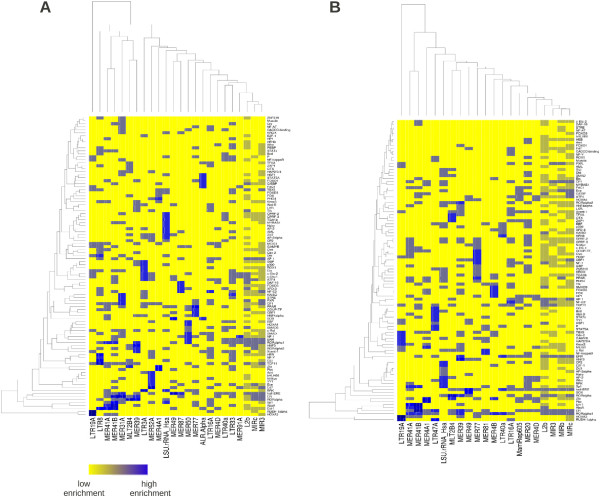
**Enriched TEs are often bound by additional TFs, besides ERα.** The heat-maps show the fraction of enriched TEs in the datasets which carry particular computationally predicted TFBSs. **A** refers to the CM dataset while **B** refers to the E2T dataset. Yellow corresponds to low enrichment fractions, blue to high enrichment fractions. In the figure we also report the hierarchical clustering of both TEs and TFs.

Remarkably enough, most of the known cofactors of ERα listed above have a TAS value above threshold in at least one of the enriched TEs (in most cases in several TEs). Moreover the same procedure allowed us to identify a few combinatorial patterns associated to selected classes of TEs, thus supporting our suggestion of a TEs driven combinatorial organization of transcriptional regulation. These data (reported in Additional file 2: Table S3 and S4 and in Figure 4) allowed also the identification of several other putative cofactors (like AP-2 factors or ERR) for which, as far as we know, experimental evidences like those mentioned above for FoxA1, RARα, GATA3, SP1 and AP1 are still missing. In Figure 4 we report the heat-maps showing the fraction of enriched transposable elements in the datasets which carry given computationally predicted transcription factor binding sites. Yellow corresponds to low fractions, blue to high fractions. In the figure we also report the hierarchical clustering of both TEs and TFs. These heat-maps are probably the best representation of the “Transposon code” mentioned above: each column corresponds to a different TE and the blue spots along the column to the particular combination of TFBSs brought about by that particular TE. Well defined combinatorial patterns involving TEs belonging to the same class can be recognised in the heat-maps. Their organization is made more explicit by the hierarchical clusterings reported along the sides of the heat maps. Smaller versions of these maps, involving only the most important known cofactors of ERα can be found in the Additional Material (Additional file 3: Figure S1 and Additional file 4: Figure S2).

Selection of combinatorial patterns using our “Transposon Affinity Score” has evolutionary implications. A TAS value above 10% (i.e. ten times the one expected by chance) is likely to occur only if the TE contained in its ancestral sequence one or more subsequences similar to the TFBS under study. If we accept this assumption then the TAS value measures the fraction of such ancient subsequences modified by evolution to become TFBSs (or conserved, if they were already present).

TFs enriched in the datasets, and in particular the known ERα cofactors, tend to be simultaneously present and to appear at fixed distance in the TEs that we studied.

**Figure 5:**
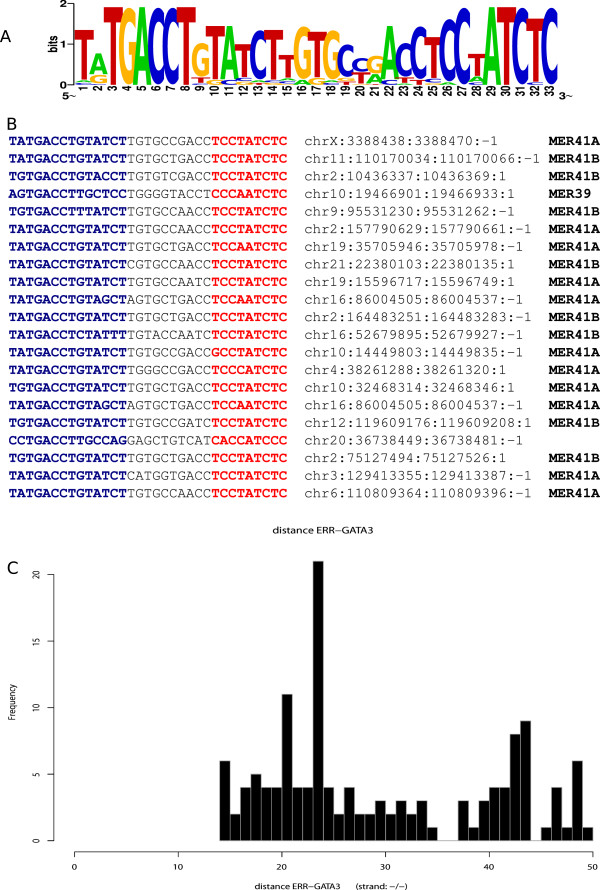
**TFBSs tend to appear at fixed distance in the TEs that we studied.** The case of MER41B is reported as an example. In **A** is shown the logo of ERα ChIP-seq regions carrying GATA-3 and ERR at the fixed distance of 24 nts (measured from start to start of these motifs). The multi-alignment of these regions is shown in **B**, together with genomic location and associated TE. **C**: distance distribution between GATA-3 and ERR in ERα ChIP-seq peaks. Notice that in all the three insets of the figure the reverse complement of the logo usually reported by Transfac is used.

A possible objection to the above statement is that simultaneous enrichment does not necessarily imply that the two TFBSs are simultaneously present in the same TE instance. To address this issue, for each set of TEs we looked for correlators of TFBSs at fixed distance along the sequence, i.e. we counted how many times a pair of TFBSs occurs exactly at a distance *d* along the TE sequence. We report the results of this analysis in Additional file 2: Table S5. An interesting example is plotted in Figure 5 for the case of ERR and GATA-3. As can be seen from Figure 5, this correlation function has a very sharp peak at a fixed distance. This behaviour is typical of most of the identified correlators and agrees with a set of recent observations (see for instance [48]) showing that TFs cooperation, to be effective, requires tight spacing patterns between the TFs.

Both in the CM and in the E2T datasets a putative ERR binding site is associated to GATA-3 at a fixed distance of 24 bp in the context of MER41A/B TEs. These TEs also host other important correlations: for example BRCA1 with E2F-1 (at a fixed distance of 33 bps) and RARα with GATA-3 (at a fixed distance of 19 bps, see the results listed in Additional file 2: Table S5). Thus we see that two of the TEs showing the highest level of enrichment in our datasets host a collection of TFBSs located at a fixed distance among them, whose relative position seems to be carefully conserved by evolution. This example is interesting also for another reason. BRCA1 is characterized by a PWM with a very low information content and for this reason we could not single it out in the enrichment analysis described in the previous section. The same happened also for other TFs with similar scarcely informative PWMs. This problem is completely overcome by the correlation analysis discussed here, which is much more constrained and allows precise identification of TFs even when they display scarcely informative PWMs.

### The genes of our datasets regulated by particular classes of TEs show a non trivial enrichment with respect to a few Gene Ontology categories

To further support these results, we searched if genes connected to specific TEs (or classes of TEs) are enriched for specific GO categories, following the procedure discussed in the Methods section. We performed this analysis both on the whole set of enriched TEs and separately on the subset of TEs conserved between human and mouse. Results of the analysis are reported in Additional file 2: Tables S1 and S2 for the whole set of enriched TEs and in Additional file 2: Tables S8 and S9 for the subset of conserved TEs.

We found several interesting results. A few of them are clearly related to the expected function of estrogen, like “cell-cell signaling” (p = 4 10^-3) or “secreted” (p = 5 10^-4), (enriched as expected in the E2T dataset) but a few others, which present rather strong functional enrichment are new and could suggest new functions (both in CM and E2T conditions) driven by selected classes of TEs. Interesting examples are GTPase regulator activity (p = 6 × 10^-5), regulation of Ras protein signal transduction (p = 6 × 10^-4) or Keratin (p = 10^-6). In particular, this latter category, which is enriched in the E2T dataset and is strongly associated to the MER20 TE, suggests that this particular class of TEs could play a critical role in remodeling cell morphogenesis, a key process in mammary gland differentiation [49]. In agreement with this observation, it is very interesting to notice that this specific TE is enriched in E2-treated dataset suggesting a link with increasing level of estrogen during pregnancy.

## Discussion

### General considerations on the regulatory role of TEs

There are by now several evidences of the important regulatory role played by TEs. For instance in [50-53] a few examples of TEs playing the role of enhancers are discussed. In [54-57] the authors showed that Alu [55,56] and Line [54] are enriched in TF binding sequences. The authors of [57] using the FANTOM4 data found more than 20000 candidate, TE-derived regulatory regions. More recently one group [58] discussed the role of TEs in organizing and modeling CTCF binding in Mammals and others [59] have shown the central role of MER20 to associate with TFs that control gene regulatory network for pregnancy in placental Mammals.

Given the importance played in our analysis by these classes of TEs, we shall concentrate here in particular on the evidences supporting the widespread regulatory role of MIR-like and ERV-like transposons.

### MIR-like Transposons, which are specifically enriched in E2T cells, play an important regulatory role in Mammals

A few recent results strongly support the idea that MIR-like transposons should play important roles in gene regulation in Mammals.

it was shown in [17] that the MIRb family is overrepresented in mammalian CNEs (conserved noncoding elements).

a TE belonging to the MIRb family was shown to play a crucial regulatory role in the Reelin signalling pathway, which allows neurons to complete their migration in the developing brain [17].

a set of MIR3 and MIRb TEs (conserved between human and mouse) were demonstrated to regulate immune response genes [60].

MIR3 and MIRb (which are both highly enriched in our E2T dataset) share a common core of 70 bps which is strongly conserved in human and mouse [28,61].

It was suggested a few years ago that this remarkable conservation could have a regulatory origin and that this shared 70 bps core could contain the binding sequences of several DNA binding proteins [26,60]. Our results suggest that ERα could be one of these TFs.

To test this hypothesis we aligned all the instances of this conserved core in the MIR-like TEs that we found in the two datasets. Then we analyzed the aligned “core” sequences using both Jaspar and Transfac binding matrices. We found at the very beginning of the core sequence a high score half-ERE, followed in many cases by other instances of the same binding sequence.

Moreover in a few cases we found examples of point mutations with respect to the reference core sequence (like the A -- > G mutation in the 14^th^ position of the core sequence) which led to the creation of additional high score half-ERE sequences. These point mutations seem to be positively selected in the dataset (they occur much more often that the other possible point mutations). The results of the analysis are reported in Additional file 5: Figure S3, in which we report in the top line the reference core sequence (as reported in [26]) and then list in each of the following lines one of the instances of the core that we found in our ChIP-seq datasets. For each of them we report in bold face the nucleotides which are conserved with respect to the original core sequence; in red colour the half-ERE binding site, in blue colour the AP1 binding site and green is for RORalpha binding site.

### Examples of exaptation of ERV-like TEs

ERV-like TEs are probably the class with the largest number of known exapted instances with regulatory function*.* At the same time ERV-like TEs are known to be preferential targets of several important TFs. For instance, it was shown in [12] that ERV1 is the class of TEs with the highest affinity for OCT4, NanoG. In this respect our analysis suggests that ERα and a few ERα related TFs should be added to this list. This idea is also supported by the analysis of the distance from the TSS of the nearest genes reported in Figure 3 showing that ERV-like TEs targetd by ERα are preferentially located in the vicinity of the TSS and are thus likely to have a regulatory role.

An interesting feature of ERV-like TEs is that, since the ERV expansion occurred in a rather large time window, there are several examples of primate specific ERVs. This represents a major difference between ERV-driven and MIR-driven regulation. A list of examples of ERV exaptation as promoters can be found in [62]. All the examples listed in [62] are not present in mouse and therefore are expected to have been exapted in the last 100 Myrs.

From these examples a few general considerations emerge:

ERV-like TEs are usually exapted as alternative promoters, their main role being to ensure tissue specifity of gene expression [57].

In several cases after exaptation the ERV-derived promoters become the primary promoter.

In most cases these new promoters have only moderate effects on gene expression. A notable exception to this trend is represented by placental genes. In these cases ERV-derived promoters have a very strong effect and are essential for the expression of the regulated genes in the placenta [63].

As for the function of these ERV-derived promoters, it appears that they play a particularly important role in the regulation of placental development [62], an observation in good agreement with our analysis, showing that ERV-like TEs are enriched in ERα and ERα-related TFBSs.

Another very interesting and nontrivial example is given by prolactin regulation. A genomic instance of the MER39 TE (which is enriched in both our datasets) regulates the expression of endometrial prolactin which is essential for pregnancy both in rodents and in human in a primate specific way. Remarkably enough the role played by this MER39 in human is played by another (similar) TE (i.e.: MER77) in rodents (notice that also MER77 is in our list of enriched TEs). We see in this example the competition between rewiring and evolutionary pressure which has driven the exaptation of different but functionally similar TEs in different species leading to this very nice example of convergent evolution [64].

### Our results support a driving role of TEs in the appearance of combinatorial regulation in complex Eukaryotes

We have shown that a few classes of transposons are strongly enriched among the ERα targets and that these host the binding sequences of several known interactors of ERα. From these observations emerges that TEs played a crucial role in the evolution of the regulatory network of higher Eukaryotes, not only by rewiring the network, as already observed in various papers [8,12,19,20], but also by allowing an easy implementation of the combinatorial rules. The emerging paradigm is that TEs may be considered as templates of pre-organized combinations of binding sequences which can then move around in the genome carrying an already prepackaged combinatorial regulatory information which can subsequently be fine tuned by local evolutionary moves (mainly point mutations) according to the specific needs of the target genes.

All these considerations support the idea that particular classes of repetitive features had a central role in the diffusion of specific combinatorial patterns of regulation on a genomic scale. This idea nicely agrees with the recent observation that waves of TEs expansions (predating the mammalian radiation) had a central role in modeling CTCF binding location in mammals [58]. This is coherent also with the work of [59] who have shown that MER20 contributed in a significant way to create the gene regulatory network important for embryo implantation key events of placental mammals. In our study we have found that MER20, enriched to ERα binding site upon estrogen treatment, is near keratin cluster genes suggesting that this specific TE class may play a role in mammary gland differentiation, in addition to placental development.

Pushing these considerations to their extreme to consequences, it is tempting to guess that combinatorial regulation was actually *made possible* by the appearance of transposable elements which had a *driving role* in the creation and organization of the combinatorial scheme of transcriptional regulation which we observe today.

At the same time our analysis suggests that when a specific association between a TF and a class of transposons exists, then the evolutionary success of this TF could be related to the evolutionary success of the associated transposon. In this respect the association between Estrogen Receptor and MIR-like transposons represents a perfect example.

A TF very similar to the Estrogen Receptor (the “ancestral steroid receptor”) exists also in very distant species like mollusks [65], however in these organisms it has a very limited role. It is only at the beginning of the mammalian radiation that this ancestral steroid receptor underwent a set of duplications, giving rise to all the family of mammalian steroid receptors and their role became essential in mammalian development and reproduction. It is tempting to relate this impressive evolutionary success with the simultaneous expansion of MIR-like transposons which, as we have shown here, are preferential targets of ERα and ERα-related TFs.

It is important to stress that in the case of ERα (as for other nuclear receptors) a crucial condition for the success mentioned above was the evolution of the ligand-binding ability. The origin of this ability is still a debated issue [66,67] with two main opposing hypothesis “the ancestral receptor” model and the “ancestral orphan” one. In this respect it is interesting to notice that MIR-like transposons are especially enriched in particular in the E2T dataset, which corresponds to acute exposure to estrogen where ER-binding intensity increases. Even if this observation by itself does not allow to distinguish between the two scenarios, it shows that transcriptional specificity for MIR-like TEs and ligand-binding ability are deeply connected (E2-bound ERα preferentially binds MIR-like TEs) and suggests that the appearance of MIR-like transposons could be exactly the event allowing to combine the two abilities and at the same time to drive the genome-wide expansion of ERα transcriptional activity.

A possible objection to this picture is that, even if statistically enriched with respect to a random reshuffling, the overall number of MIR-like peaks in our datasets is only a small portion of the total number of peaks (7.3% in the CM case and 10.2% in the E2T case). It is not obvious that they could really influence the functional evolution of ERα. Indeed it is well possible that other evolutionary mechanisms, still to be understood, played a role together with MIR-like TEs in shaping the functions that the Estrogen Receptor has acquired in these last 500 Myrs. However it is important to notice that the above percentages increase to higher values if we consider the fraction of ChIP-seq peaks containing simultaneously ERα and one or more of its most important cofactors, like FoxA1, RARα or AP1 (see the heatmap in Figure 4). This agrees with the idea that combinatorial organization of the regulatory interactions made ERα evolving its functions and that this combinatorial organization was more likely mediated by TEs.

It would be very interesting to extend these observations to other classes of TEs (in particular to Alus whose important regulatory role has been already noticed in [55] ) and to other TFs. It is becoming increasingly clear that TFs can be classified as a function of their age and that in some cases they acquired their regulatory role in a precise time window of the evolutionary process. It would be interesting to test if also in this case it is possible to associate these TFs to precise transposon classes and to relate their evolutionary success to the expansion of the associated transposon class.

## Conclusions

In this work we sought to better understand the role of transposable elements in shaping the regulatory network of higher Eukaryotes. Several papers appeared in these last years on this topic and it is by now well understood that TEs contributed to a substantial rewiring of the regulatory network of complex Eukaryotes. In this work we add another piece of information to the picture looking at the role of TEs in the emergence and diffusion of combinatorial transcriptional regulation. The rationale behind this study is that while it is easy to create a single binding sequence by point mutation it is difficult to understand how a local evolutionary process could create an extended (non-local) combination of binding sequences. A possible solution is to assume that a suitable template for the sought combination of motifs already exists in the genome and is then transferred as one in the regulatory region of the target genes. To test this idea we focused on a particular transcription factor: the Estrogen Receptor alpha (ERα) and, using two different sets of ChIP-seq data, performed a set of enrichment tests on the TE distribution within the datasets. We found that ERα preferentially targets a well defined set of TEs and that these TEs host combinations of transcriptional regulators involving several of known co-regulators of ERα. Moreover, a significant number of these TEs turned out to be conserved between human and mouse and to be located in the vicinity (and thus candidate to be regulators) of important estrogen-related genes.

## Methods

### Datasets

We used two whole-genome occupancy datasets. The first one is the dataset published in [24] (“Complete Medium” (CM) dataset in the following) derives from MCF7 cells cultured in complete medium, i.e. DMEM added of 10% Fetal Calf Serum. Data are available at http://genesdev.cshlp.org/content/24/2/171/suppl/DC1 (“Supp_Data_Binding_Files.xls”). The authors define this culture as “proliferating cells” and the concentration of active estrogen in media can be estimated from 0.01 to 0.1 nM. The second one is the dataset published in [25] (“E2- treated” (E2T) dataset in the following) derives from MCF7 cells cultured for 3 days in hormone-deprived medium (phenol red-free DMEM/F12 containing 5% charcoal-stripped FBS) and then treated with 100nM estradiol for 45 min. Data are available at http://www.nature.com/msb/journal/v6/n1/suppinfo/msb2010109_S1.html in the folder “Supplementary_Dataset_1”, in a file named “Suppl_combined_table_MCF_T47D_ER_binding_by_ChIPseq_hg18.xls” (we considered only peaks from MCF7 cells). In both experiments ChIP-seq library was obtained using Illumina Solexa ChIP-seq sample processing methods.

The two datasets have a comparable number of entries: 14505 for the E2T dataset and 16043 for the CM one. This similarity will simplify the statistical analysis in the following and will allow us to reliably compare the results obtained in each dataset.

A remarkable feature of the datasets is that, despite the differences in culture and treatment, their intersection is very high: 9545 peaks out of the 14505 from “E2T” dataset have at least one base overlapped with 9924 peaks out of 16043 from “CM” dataset. (the two numbers do not coincide due to multiple intersections of nearby peaks).

### Identification of Transposable Elements

We used the Ensembl [31] database annotation of transposable elements, which is based on the RepeatMasker database [68,69]. Correspondence between single transposable elements and classes was also taken from Ensembl.

### Enrichment tests

Following [12] for each peak declared in the given dataset, we extracted a 200 bp DNA-wide window centered on the middle of the peak.

Then we generated 1000 datasets composed by randomly chosen peaks, each containing the same number of regions with the same length (200 bps) and the same nucleotide distribution as true regions. To do this, we divided each chromosome in genomic windows of 1,000,000 bps and, for every real peak, the corresponding random peak was taken with flat distribution from the same window.

We then annotated all repeats falling in the peaks belonging to the real dataset and in the 1000 datasets of random peaks. This way we managed to obtain, for each transposable element, mean and variance, which we used to calculate a *z-score* z_r_ = (x_r_ − μ_r_) / √s_r_, where x_r_ is the occurrence of a particular transposable element *r* in the original dataset, while μ_r_ and s_r_ are respectively its mean occurrence and its variance in the 1000 random sets.

We performed the same analysis also with classes of transposable elements and similarly calculated the z-score z_c_ = (x_c_ − μ_c_) / √s_c_, where x_c_ is the occurrence of a particular class of transposable elements in the original dataset, while μ_c_ and s_c_ are respectively its mean occurrence and its variance in the 1000 random sets.

We carefully tested that our results were robust with respect to different choices of this window size (data not shown).

### Functional enrichment analysis

We associated putative regulated genes to the ChIP-seq peaks of our datasets as follows. For each Ensembl transcript we choose a window of +/− 20Kbp around the transcription start site (TSS). If inside one of these windows we find a peak of our dataset we assume that the gene to which the transcript belongs is regulated by ERα binding to the selected peak. We chose this particular window size since in [23] it was shown to be an optimal window to identify ER-binding events in target genes. Also in this case we verified that our enrichment results were robust against different choices of the window size.

This way we could associate to each TE a set of putative regulated genes looking for all the genes regulated by peaks belonging to a particular instance of that TE.

For each of these sets we performed a functional enrichment analysis using the DAVID Bioinformatics Tools [70] and choosing as background model the set of all the regulated genes in our dataset.

### Identification of putative ERα interactors, Transposon Affinity Score, TFBS correlators and the definition of the ER PWM

We scanned for the presence of TFBSs within a window of 200 bps centered in the middle of the ChIP-seq peaks using all the position-specific scoring matrices from TRANSFAC professional 11.2.

Then, for each TE we computed the fraction of instances in the dataset carrying particular TFs. This quantity, which we shall denote in the following as Transposon Affinity Score (TAS), takes values in the range [0–1] and can be used as an indicator of the specific affinity of a class of TE elements for a particular TF. Scan thresholds vary according to information content of PWMs and were chosen for each TF so as to find a TAS value of 0.01 for a random sequence (see Additional file 1: Additional Material for details)

We then set an enrichment threshold of 10% in this TAS value. The TFs with a TAS above this threshold in at least one TE class represent the list of our putative ERα interactors.

We used the same scanning procedure discussed above to identify correlators of TFBSs at fixed distance along TEs, i.e. enriched motif spacing patterns for given TEs. The rationale behind this analysis is that probably it allows TFs to optimally interact with each other.

Among the PWMs that we studied a particular role is obviously played by the PWM of the Estrogen Receptor. In our analysis we considered both the whole Estrogen Receptor PWM (denoted in Transfac as ER) and the half-site of estrogen response element (denoted in Transfac as half-ERE). The corresponding logos are reported in the Additional Material.

## Abbreviations

CNE: Conserved Non-coding Element; CM: Complete Medium; E2T: E2 Treated; ER: Estrogen Receptor; ERE: Estrogen Receptor Element; ERR: Estrogen Receptor Related; ERV: Endogenous RetroVirus; GO: Gene Ontology; MIR: Mammalian Interspersed Repeat; PWM: Position Weight Matrix; TAS: Transposon Affinity Score; TFBS: Transcription Factor Binding Site; TE: Transposable Element; TF: Transcription Factor; TSS: Transcription Start Site.

## Competing interests

The authors declare no conflicts of interests.

## Authors’ contributions

AT implemented most of the algorithms for bioinformatic data analysis. LC, SC, OF and MDB partecipated in the biological interpretation of the results. DC supervised the bioinformatic and statistical analysis. MC designed and coordinated the project. All the authors participated in manuscript writing, read and approved the final version.

## Supplementary Material

Additional file 1 **Additional Text and Methods.** Content is divided in the following sections: List of Additional Tables, Transposable Elements Annotation, Monte Carlo Simulation, Transcription Factor Binding Sites Identification, Correlators Identification, ER logo, Half-ERE logo. Click here for file

Additional file 2 Additional Tables S1 to S11. Click here for file

Additional file 3 **Figure S1.** The heat map shows the fraction of enriched transposable elements in the CM dataset which carry particular computationally predicted transcription factor binding sites. Here only a selection of the most important known cofactors of ERα is considered. Click here for file

Additional file 4 **Figure S2.** The heat map shows the fraction of enriched transposable elements in the E2T datasets which carry particular computationally predicted transcription factor binding sites. Here only a selection of the most important known cofactors of ERα is considered. Click here for file

Additional file 5 **Figure S3.** The figure shows alignment of MIRs from both CM dataset and E2T dataset. The first line of the figure is MIR core sequence (Smit et al., 1995); the binding site of half-ERE is in red. Following there are all other MIRs. Red is for half-ERE binding site, blue is for AP1 binding site and green is for RORalpha binding site. If the character in the alignment matches the character in the core sequence, it is depicted in bold. Click here for file
