# Supplementary material for: The role of Transposable Elements in shaping the combinatorial interaction of Transcription Factors
Source: BMC Genomics. 2012 Aug 16;13:400. doi: 10.1186/1471-2164-13-400 (PMC3478180; doi:10.1186/1471-2164-13-400)
Supplement: Additional file 1 — Additional Text and Methods. Content is divided in the following sections: List of Additional Tables, Transposable Elements Annotation, Monte Carlo Simulation, Transcription Factor Binding Sites Identification, Correlators Identification, ER logo, Half-ERE logo. [file 1471-2164-13-400-S1.pdf]

The role of Transposable Elements in shaping the combinatorial interaction of Transcription Factors

Additional Online Material

Testori A. [1,3,±], Caizzi L. [1,2], Cutrupi S. [1,5], Friard O. [1], De Bortoli M. [1,3], Corà D. [1,4,*,±] and Caselle M.[1,6,*,±]

(1) Center for Molecular Systems Biology, University of Turin,
c/o IRCC - Str. Prov. 142 Km. 3.95, I-10060 Candiolo, Turin, Italy;

(2) Bioindustry Park Silvano Fumero,
Colleretto Giacosa, Italy;

(3) Dept. Oncological Sciences, University of Turin,
Str. Prov. 142 Km. 3.95, I-10060 Candiolo, Turin, Italy

(4) Systems Biology Lab, Institute for Cancer Research and Treatment (IRCC),
Str. Prov. 142 Km. 3.95, I-10060 Candiolo, Turin, Italy

(5) Dept. of Life Sciences and Systems Biology, University of Turin,
v. Acc. Albertina 13, 10123 Turin;

(6) Dept. of Physics, University of Turin,
v. P. Giuria 1 - I-10125 Turin, Italy;

(*) = These authors contributed equally to this work as senior authors.
(±) = To whom correspondence should be addressed.

Email: alessandro.testori@ircc.it,
davide.cora@ircc.it,
caselle@to.infn.it.

Table of Contents

List of Additional Tables 2
Transposable Elements Annotation 3
Monte Carlo Simulation 3
Transcription Factor Binding Sites Identification 3
Correlators Identification 4
ER logo 4
Half-ERE logo 4

List of Additional Tables (Additional_Tables.xls)

S1. List of GO enriched categories for the subset of TEs (or classes of TEs) enriched within the CM dataset. Only GO categories with a p-value lower than 0.01 are reported.

S2. Same as Table S1 but for the E2T dataset.

S3. TAS values for the CM dataset. Each line corresponds to one of the enriched TEs that we found in the dataset. Each column corresponds to one of Transfac PWMs. Only those which go over the threshold of 0.1 in at least one TE family are reported in the table. These are the data used as input for Figure 4A in the main text.

S4. Same as Table S3, but for the E2T dataset. These are the data used as input for Figure 4B in the main text.

S5. Correlators at fixed distance of TFBSs. For each correlator we report the ordered pair of TFs, the relative strand (+/-) strand orientation of the two binding sequences and the relative distance. Then we list all the instances in our dataset and when they coincide with a TE we report the corresponding classification. The label partial/full indicates if the correlator is fully or only partially contained in the TE. It is interesting to notice that in a few cases these correlators are associated to more than one class of TEs. This could be the signature of a process of convergent evolution acting on these regulatory modules.

S6. List of TE from the CM dataset conserved in mouse. In the second column for each TE we report the ratio of conserved versus total instances in the dataset.

S7. Same as Table S6 but for the E2T dataset.

S8. List of GO enriched categories for the subset of TEs (or classes of TEs) conserved between human and mouse enriched within the CM dataset. Only GO categories with a p-value lower than 0.01 are reported.

S9. Same as Table S8 but for the E2T dataset.

S10. List of genes which are expected to be regulated by human-mouse conserved transposons in the CM dataset. In the first column we report the TE name and in the following columns the list of putatively regulated genes obtained following the procedure discussed in the main text (genes whose TSS occurs at a distance smaller than 20Kb from the TE).

S11. Same as Table S10 but for the E2T dataset.

Transposable Elements Annotation

The genomic annotation of transposable elements was downloaded from The Ensembl database using perl API – Ensembl Repeat annotation (method: public Listref Bio::EnsEMBL::Slice::get_all_RepeatFeatures ()). Also the TE classification (classes) was downloaded from Ensembl, via method public String Bio::EnsEMBL::RepeatConsensus::repeat_class (). For every repeat and for every class we then managed to have chromosome, start and stop: this is referred to as Repeat annotation in the following.

Monte Carlo Simulation

For every TE and for every class, we counted how many of its genomic instances fall on ChIP-seq peaks. A TE is counted if its annotation overlaps for at least one nucleotide to the 200 bp DNA-wide window (as in Kunarso *et al,* 2010) centered in the middle of the peak (200P in the following). If a TE annotation overlaps more than once within the 200P, it is counted only once.
1000 random ChIP-seq datasets were then generated for Monte-Carlo simulation, all composed by 200 bp wide peaks. Genome was divided into windows of 1,000,000 bps and every real peak was assigned to one of these windows: for every real peak the corresponding random peak was chosen from the same genomic window. We checked that random peaks, as this is true for real peaks, were not overlapping with one another. The same TE count that was done for real peaks was then done also for random peaks.

Transcription Factor Binding Sites Identification

All 200P were scanned for the presence of TFBSs using Positional-Weight-Matrices (PWMs) built from Transfac Matrix Table, Release 11.2. In the search for putative interactors of ERα, scan thresholds were selected according to information content of PWMs: it was chosen to be the minimum threshold that allows the TF to be found in less than 1% of randomly chosen 200 bp wide sequences. The two following figures show logos of estrogen related matrices we considered.

ER logo: logo was generated with Weblogo 3 (http://weblogo.berkeley.edu/).

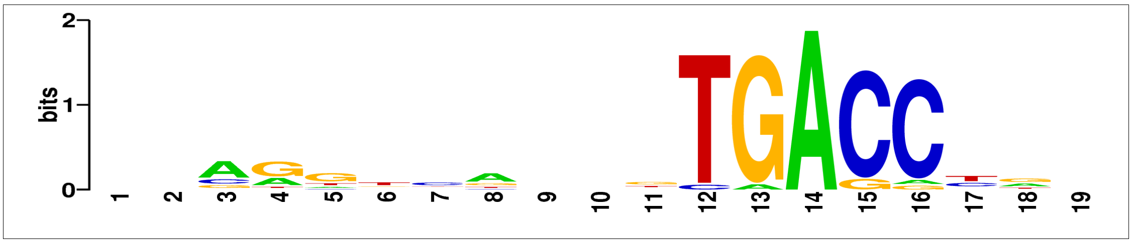

half-ERE logo. This logo was generated as in the previous figure, but the reverse complement of transfac half-ERE site matrix was considered: this to make comparison with ER logo easier.

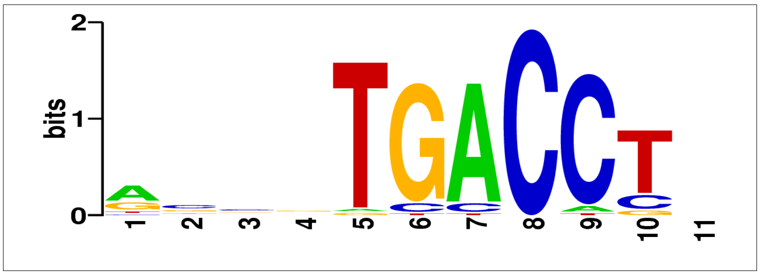

Correlators Identification

All 200P were scanned for binding sites of transcription factors (TFs) present in heat map of Figure 4 (scan threshold = 0.7). To these TFs , we added GATA-3 and BRCA1, as they are known ERα interactors. As TFs bind to DNA in a cooperative manner, we searched for enriched motif spacing (i.e.: couples of TFs, TFa and Tfb in the following) in 200P of both ChIP-seq datasets. We considered only a window of 50 bps from the start of each TF. We distinguished cases in which both TFBSs occur on the same strand from those in which TFBSs occur on opposite strands; we further indicated whether TFa happened to occur upstream or downstream with respect to TFb. In total, we have four categories of displacement: same strand / opposite strand; upstream / downstream. Statistical significance of motif spacing enrichment was evaluated as in Whitington T. *et al*, 2011.

References:

Kunarso G, Chia NY, Jeyakani J, Hwang C, Lu X, Chan YS, Ng HH, Bourque G (2010) Transposable elements have rewired the core regulatory network of human embryonic stem cells. Nat Genet. **42(7):** 631-4

Whitington T, Frith MC, Johnson J, Bailey TL (2011) Inferring transcription factor complexes from ChIP-seq data. Nucl. Acids Res. **39(15):** e98
