# Supplementary figures and images for: The role of Transposable Elements in shaping the combinatorial interaction of Transcription Factors
Source: BMC Genomics. 2012 Aug 16;13:400. doi: 10.1186/1471-2164-13-400 (PMC3478180; doi:10.1186/1471-2164-13-400)

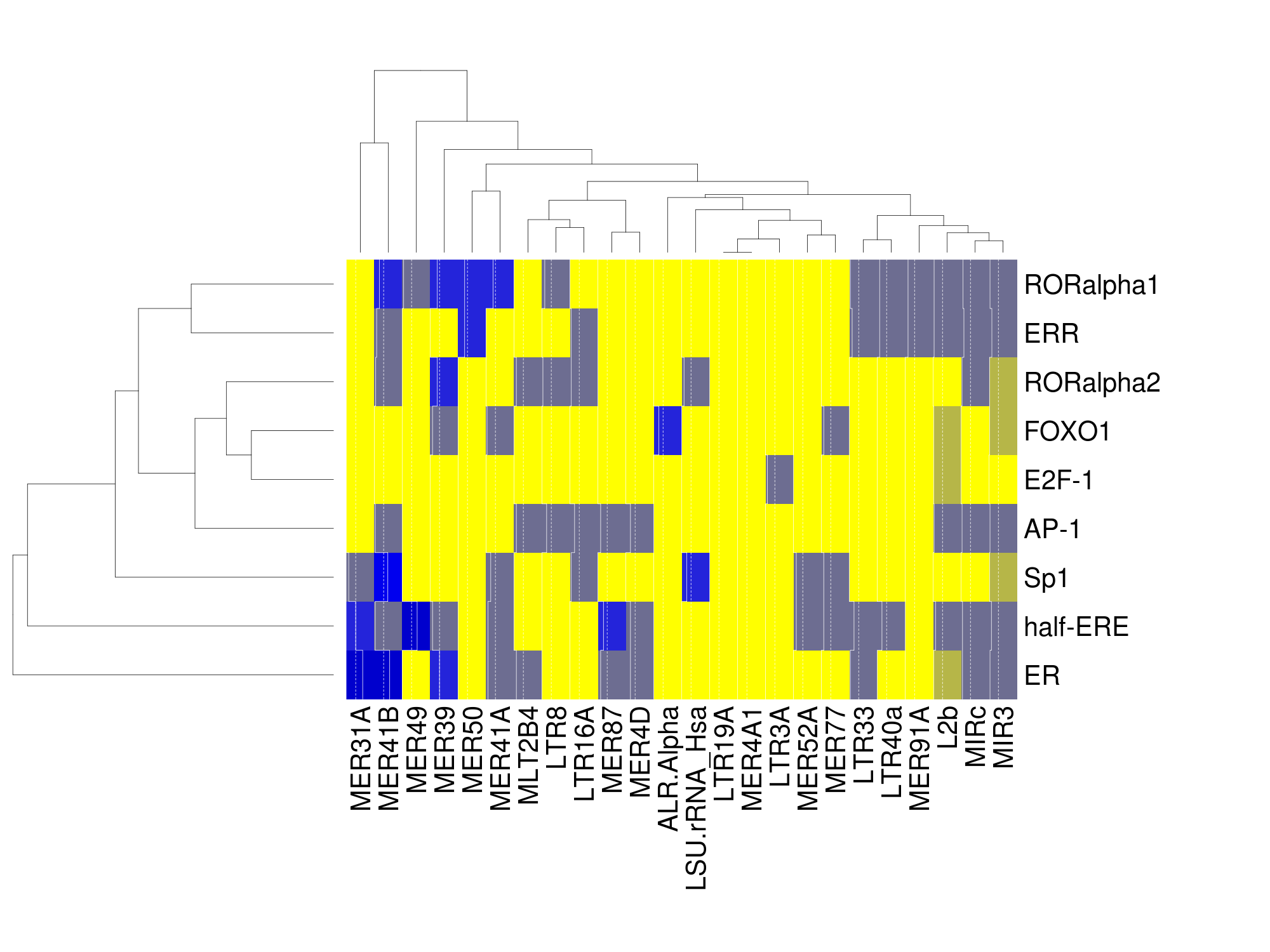

Supplement: Additional file 3 — Figure S1. The heat map shows the fraction of enriched transposable elements in the CM dataset which carry particular computationally predicted transcription factor binding sites. Here only a selection of the most important known cofactors of ERα is considered. [file 1471-2164-13-400-S3.png]

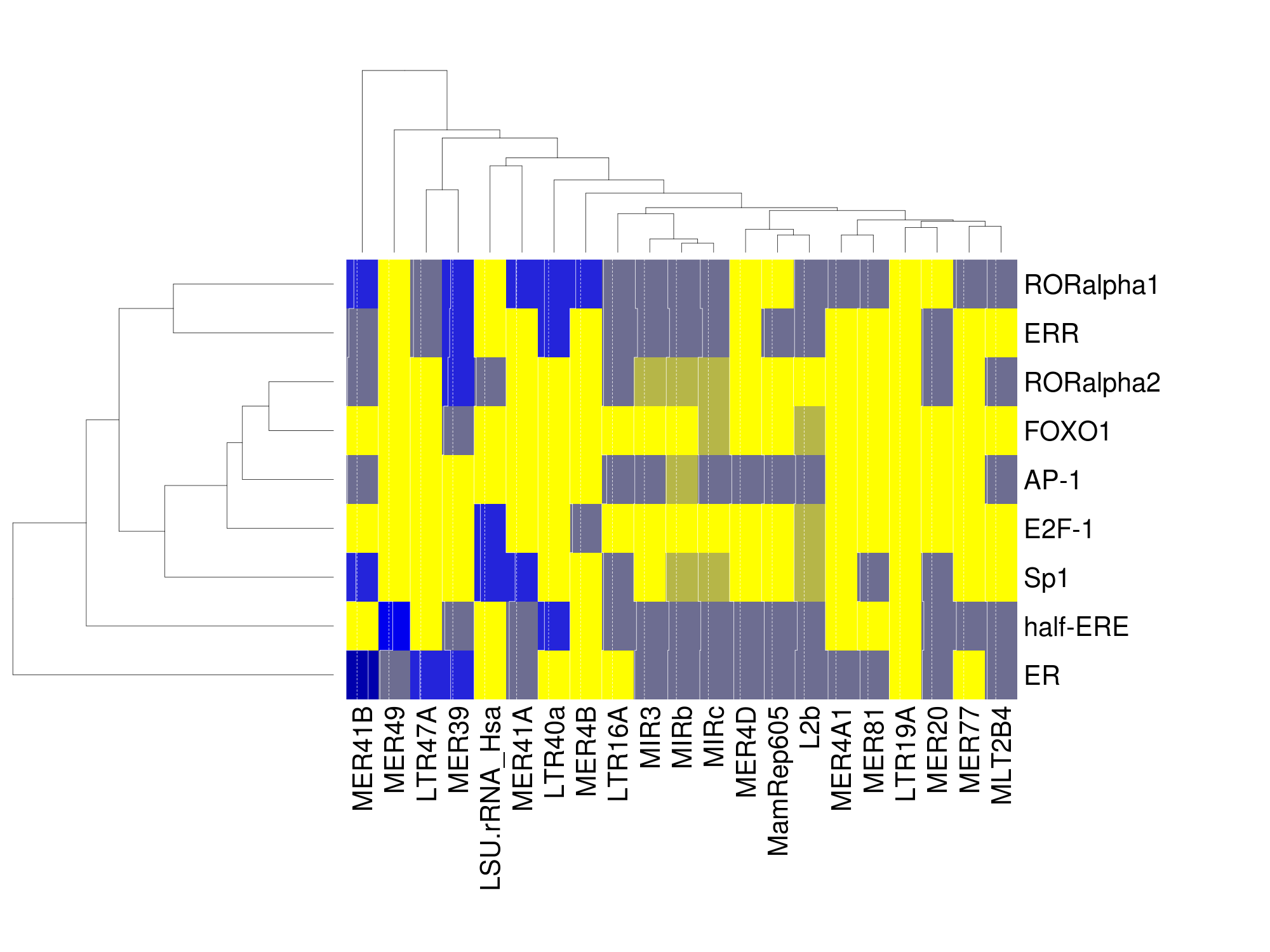

Supplement: Additional file 4 — Figure S2. The heat map shows the fraction of enriched transposable elements in the E2T datasets which carry particular computationally predicted transcription factor binding sites. Here only a selection of the most important known cofactors of ERα is considered. [file 1471-2164-13-400-S4.png]
