# Supplementary material for: The role of Transposable Elements in shaping the combinatorial interaction of Transcription Factors
Source: BMC Genomics. 2012 Aug 16;13:400. doi: 10.1186/1471-2164-13-400 (PMC3478180; doi:10.1186/1471-2164-13-400)
Supplement: Additional file 5 — Figure S3. The figure shows alignment of MIRs from both CM dataset and E2T dataset. The first line of the figure is MIR core sequence (Smit et al., 1995); the binding site of half-ERE is in red. Following there are all other MIRs. Red is for half-ERE binding site, blue is for AP1 binding site and green is for RORalpha binding site. If the character in the alignment matches the character in the core sequence, it is depicted in bold. [file 1471-2164-13-400-S5.pdf]

```
GTGACCTTGGGCAAGTTACTTAACCTCTCTGTGCCTCAGTTTCCTCATCTGTAAAATGGGGATAATA

--AAAATTGCTCAGGTCACCTTGCCTCAGTGAGCCTTGGTTTATTTACCTTTAAAATAGGGGTTATA
A-AACCTTGG-CAATCCAATGGACTTCTCTGTCCTTTACTTTTCCCAGG-GTCAAACGAGATTAAAA
---------------------AACCCGCCCCAGACTCAGTTTC-TCACTTGTGCA-TGGGGCCAGTA
AAGACTCCAGGCAAGCTG-TGGACTGGCCCATGCCTCAGTCTCCCCGACTGTTACATGCAGCAAGTG
AAGCCCTCCGGCAAGGTTTCGAACCTCTCTG----------------GCTGAGATAAGGTGATAACA
-------------AAGTATTTCACTTTTCCGGATTTCTGTTTGCT-ATCTGTAAAATGA--------
AATTCCTTGGGCAGCTCACTTTACTTCCCCAAGCCTCCATTTTCTCATCTATAAAATGAGGATAATA
ACAACTCTTGGCACGGCACT-AACCTTCCTGTGCCT--GTTTCTTGGTTAACAAAATGGGTATGATT
---------------------ACCACCCTCATGCCTCAGTTTTCCCATCTCTAAAGTGGGGGTAACA
-----------------------ACCCACTGCCCCCCAGCTTTCTCATCTACAAAACAGGGATGCTA
-----------------ACTTCACCTTTTGGGGCGTCTGTATCCTCATCTACAAAA-----ATGGTA
----------------------ACTTCTCTGCACCTCAGATTCTGCATCCATAATTGGGGGGC----
AGAACCTTG---------CTTAGCTTGTCTTTGCTTCCGTTGCCCCATATGTGAAGTGGTGATGGTA
AGAACTTAAGGCAATTCAGTTAACTTCTCTGTGCCTCGGTTCTCTCATCTGTGTAA-----ATAGGA
AGAAGACTGGG---ACTAGTTAATTTCTCCAGTGCTCAGCTTC-TTATCTACAGG-TAAGAATTGTC
AGGATCTTGGGCAAGTTTCTTA-TCTGTCTAAGCCTTGACTTCATCAGATGGAAAGTGGAGCTAATA
---------------------------------------------------------AGGGGTATTG
------------AGGTTCCTACTGCTCTCTG-GCCTCAGGCTACTCACCTGCAAAATAAAGAG----
ATAACCTTGAGAAAGTTACTTAGCCAGTCTAAATCTCATGTTCCCCTCAAGTCAAATGGGGATAATG
ATAACCTTGAGAATATT-CTTA--------------------CCTCCTCTGTCAAATGGAGACAAGA
ATAACCTTGCCTAAGTTACTTAACTCCTCTATGCCTCAGTTTCTTCATCTGTAAAACAGGGGTAACA
ATAACTTGGAGCAAGTAACTTGACCTCGCCTAGCCTTTGTTTTCTCATATTTAAAAT----ACGTCA
ATAGCCCTGGACAAAGGGTTTTGCCTGTCTGGGTCCCAGTTTTCTCA--------------------
ATCAGTTCTATAAAGTTCCTTAAGTTCTTCAGACCTCTATTTCTTCATCTCGAAAACTGGGAGAAT-
ATGA------------------ACCATTCTGAGCCTTAGTTTGCTCTTCTATAAACCGGGGAGAGCA
ATGACATAGGGCAAATTGCTTGACCTCTCTGAGCCTCAGTGTCATCACCCACAAA-TGGGCATGGCA
ATGACCCTAAATCGGACATTTACAATTTCTT------------------------------------
ATGACCCTGAGCAAGTCCCTTCTCTGCCCTGGGCCTCAGTCTCCCCATCTATAATAGG---------
ATGACCCTGGCCAGCTCCCTCC---------------------------------------------
ATGACCCTGGGCAAGTTGCCCCACTTCGGCGAGACT--GTTTCCTCA-CTATAAAATAGTGGCAATT
ATGACCTCAGGCCAGCCACTTTAACTCTCAGTGCCTCA-TTTCTCTAACTTTAAAATA---------
ATGACCT-GGGCTAGCGAGGGAAGCTGTCTAGGTCTTTGTTTTCTGGTCTG-GGAATGGGTTTGATG
ATGACCTTAGGCCAGTTGTTTAA--TTTTTGTGACTCAGCTTCTTCTTC----ATGTCAGTTCTATC
ATGACCTTAGGTAAGATA-------------------------------------------------
ATGACCTTGAATAGGTCAGCTAAACTTTCCAGGCCTC------------------------------
ATGACCTTGAGTACAT--ATGCCCTTCCCTAGGCTTCAATCTCTCAATCTATGAAACAAGGA-----
ATGACCTTGCCAAGGT--CTGGACCTCT--GTGCCTCAGTTTCCTCAGGTATAAAATGGAGGTAAGG
ATGACCTTGGGCAAGTGACCTGTCCTCTTGGGACCTGATTTGCCCCAACTGCAAAGTG---------
ATGACCTTGGGCAAGTTGCCCAGTCTCCCTGT-----------------------------------
ATGACCTTGGGCA--TTATTTGACCTGTTTA--CTGCAGTTTCCTCCTGTGTAAAATGGGGATAGTT
ATGACCTTGGGTAAGCGG---ATCCCTTAAATTCC--------------------------------
ATGACCTTG-----TTAATTTTCTCTCTCTGTACCTCAGTTTC-TCAT-------------GTGAAA
ATGACTTTAGGCGAACTACTTAGCCTTT-TAAGTCTCAGTTTCTTCTTCAAGGAAAATGTGACAAAG
ATGACTTTGAGCT-GTTACTTA-----------CCTCAATTTTCTTATCTCTGAAATGGAGATCACT
ATGACTTTGTGTAAGTCATTAAACTTCC--AAAGTTGAGTTTTCTCATACATACAATGGGGAT----
ATGATCTTGGGCATATTACTTAACCTCCCTGTGCCTCAGTCTCCTCATCTGTCAAACAGGAATGACA
ATGATCTTGTGTAAGT--CTCCACCTCTCCACGCCTCAGTTTCCTCGTCCGAAAAACAAGGATGTTA
ATGATGTTGGGCAAGTTATTTAACCTCACTGTGCTTTAATTTCCTTTCCTGTGAAA-AAAGATAATC
ATGCCCTTGGGCCA--TACTT----CCTCTGCGCCTCAGCTT-------------------------
ATG-CGTTGTGCAAGTGACTGAGCCTTGCTTTGCCTCA---------TCTGATAAATGGGG------
ATGGACTTGACTGAGTTAGGAACCCTCTCTGGACCTTCATTTCTTCATATGTAAA-TGAGAATGCAT
ATGGCCCTCATCGAGGCCCTTCCCCTCTCTGGACCTCAGTTTC-TCAT-TGCAGGATGAGAAGATCA
ATGGCCTGAGGAAAGCGACCCAGCCTCTCTGACACTCAGCGTCCTGAGCTGTGAAGTGGTGGTGGGA
ATGGCCTTGGACAGGTGGCTTCACGTCTCTGGGCCTCAGGTGCCTCCTGTCT---------------
ATGTCCCTGGGTAGGTCACTTC-CCTCTCCAGGCCTC--CTTCCCCATCGGGTCAAT----------
ATGTCCTCATCTCA--TACCCAACCTCTCTGGACCTTGATCTCCTCCTTTGTAAAACAGAGATAATG
```

```
ATTACTTGGGAGAAGTTACTTGACCTCTTTGGGCCTCAGTTTCCTCACCCATAAAGTGGGGAAAGTA
------------------------------------CAGCTTCCTCCTTCTCTAAACCGGAATAA--
------------------------------------CATTTTCCTCATGTGTAAA-TGGGGGCAATA
----CCATAGCCAAGTTGCTTCAACTCT--GCATATCCATTTTCTTATCTATGACATGAAGGTATTA
----------------------------------CCCCATTTCCTCACCTGTGAAATGAGAATAGTA
------------------------CCCCCTGGCCCTCAGCTTCTTCATCTATAAGATGAAG-TGGT-
----CCCTGGATGCCTTCCTG---TCTTCTGTGC-TCAGGCTTTCCAGCTGGAAAATGGGAA-----
CCGGCCTCAG-CGGGCTGGCTGACCTCTCGGTGCCTTGGCTTCATCACCCGTAAAACAGGGAGAACC
----CCTCAGGCAAGTTACTTAACCT-TACATGCCTCAGTTTTCTCATCTGGAAAATGAGAAC----
-----------------------CCTCTCCATGCCTTGGTGCCCGCATCAGTAAAATGGTGACAATA
-----CCTGGGCAAGC-GCTTTACCTCTCTGTGCCTCGGTTTGCTCATCTGGGAATTGGGGATGTTG
---------------------------CCTGGGCCTTAGGTTGTTCAACCGTAAAATGTGGTTAACA
----CCTTGGACAAGTGAGTTTCCCTCTCCGTGCCTCAGTTTCCTCATTTGGGAAATGGGAATAATG
----CCTTGGAC--CTTCCTCTATCCTACCATGTCTTGGTTTCCTCACTTTAAAAATGGGGATATAG
---------------------------------CCTTGGCTCCCCTATCTCTATAGTGTGGACAT--
----CCTTGGGCAAATTGTCTAACCTCCCTGCGCCTCTGTTTTAGTTTCTGTACAGTGGA------A
----CCTTGGGCAAGTTACATAATCTCTCAAGACCT--GTTTCTTCATCTCCAAAATGAGAGTAATA
CCTTTCTTAAGTAAGTTA---ACCTCTTCTAAGCCTCATTTTCTCCACATGTAAAATGGGAA-AACA
-------CGGGCAAGTTACTGAATTTCTCTGCACTTTTCTTTTCGCATCTGTAAAATGGGGATACTA
CTAAGCTTTGATGAGTGATTTAACCTCTCTGTGCTTGA--TTTCTCATCTGTATAATGGGTGTA-TA
-----CTCACAATGTTTACATAACCTCTCTGTGTTACAGTTTCCTTAGCTGCAAAGTGTAGATAGAA
----------------------------------CTCAGTT--CTGGTCTTTACGCTTCAGGTCATG
-----------------------CTCCTCCGTCCCTCAGCTTCCTCATCTGTAAAATGGGGAAAACA
----------------------------------CTCTGTTGGTTCCCTCTGAAAGTGCTGATAATG
CTGACCT-ACAAAACTGACCTGATCTTTCTGAGTTCC--TTTCCTCACCCATAGAATGAGT------
CTGACCTCTGGCAA-----------------------------------------------------
CTGACCTTGAGAGAGTGGGTTGGCCTCTCTGTGCTTCATTTTCTTTGCTTCTAAAATGGAGGTAATA
CTGACTT--GGCAGGCAACTTCACCTGT-TGTGCTTCCGTTTTC-CATAGATAAGATG---------
CTGACTTTGAACAAGCCACTTAACATTTTCATGCTACATCTTCCACAAATTTAAAATGAGGATCATA
CTGAGGTCTTATAAGCTGTGTAACCCCC---TGCCTCAGTTTTCCCATCTGTAGA-TGGGGACCTCT
CTGATATTGGCCAAGTTCCCTAACCTCACCTTGCTGCAGTTTTCCCATCGGCAAAA---GAATGATA
CTGATCTTGTGTGAGTTCCTTAACCTCCCCAAACCT--GTTTCCTTACCTGCACAATGGGGGTGAGA
CTGGATTCAAACCC-TAACCTCACCTTTCTTGGCATCAGTTTCCTCATCTGTAAAATGGG-ATAATG
CTGGCCTTGGGCAGTGTCTGTGGCCTCCCTGAGTCACAGTGACCTCCACTGTACACTGGGGAGAAGA
CTGGCTGAGGCCAAGTGACTTAATCTCTAAAACCTTCA---TCTTAACTTGTACAATGTATATTAAA
----------------------------CTGTGCCTCCACTTGCTCCTCTGTGAAAGGG----AGTG
------------------------------------CTGTTTTCCTGTTTCTAAAAAGGAGATAATG
-----CTTGAGCAAGTGACTTC-CTTCTCTGGGGCTCAG----------------------------
----CTTTAGGCCCATCAACCAGTTCCTCTGTGCCTCCGTGTCCTTATTTCTAAGAGGGTAATGATG
----CTTTGGGCAAATTGCTTAA--------TGTCTTACCTTCGGTTTCTTTAGAACTG--------
--------------------------CTTTGGGCTTCAGCGCCCTCACATGTGAAATGGGGATGACA
GAAACCTTGCACAAGTCCTTTAACCTTTCTACGTCTTAAA--CCACATCTGTAAAACAACAATAATA
GAAAGCTTTCAAAAACCACTTGACCTCTCAGTGCCTTAGTTCCCTCACCTGTGAAATGGGGATAACA
G--ACACTAGCTGTGTCACTTAACCTCTC-ATGTCCCAGTTTTCTCATCTGTAAAATGGGAATGACC
GACACTCCAGGCAGGTTATTTAAATTATCTGTACCCTAATTCCCTCATCTGTAAAATGGCAATAAAA
------------------------------------------------------------GACAGTA
--GACCATGAGCCACCTTCTTGACCATTCT--GCTTCAGTGTCCTCGTGTGTAAAACGAGAAAAATA
-------------------------------GACCTCTGTTTTCTCATCTG-AAAATAAGGAAGTT-
G--ACCTTAGGCCAGTTTCTTAACCTCTGT-TGAGTTTGTTTCCTCA------------GAATAATA
--GACCTTGTGCAGGCCACTTAACCTCTCTGTGCCTCAGTTTCCCCAGCTGTGATGTGAGGTTAATA
GACTCCTTGGGCAACTTGTCTCCCTTCTCCAAATCTCAGTTTGCTCAGCTGGAAAATAGAGATGCTA
--GACTGTGGGT--GTCAGTTGAGCTGGCTGTGCCT--ATTTCCTCATCGGGGAAACAAGAGTCATT
GAGACC-------------------------------------------------------------
GAGACCTAATGGACGCTGCCCAACCACCCTGTGCCTCAGGGTCCTTGTAGGTAAAGTGAGGATAACG
GAGACCTAGGGCAAATCAGCTAACCTCTCTGTGTCTCCATTTCCCGATCTGTGAAATAAGGTCAATA
GAGACCTTAGGCAAAGCACTCCACCTCCGGGAGCCTCATTTTCCTCCACTG----------------
GAGACGTGTGGCAAAT-----GACCTCCCTG-GCCT--GTTTTCTCTTCTATGAAATGAGACT----
GAGAGCCTGGGCG---TGCTTAACCCATCCACACCTCAATTTCCTCATCTG-AAAATGGGGATGATA
```

GAGATCTTAG-CAGAGTGATTCACCCCTCCAGGCCTTGGTTTCCTTTTGAGTACAATGAGAGTG---
GAGCCTTTGGTCAAGTTATTTAATCTCTGTCAACATCAAT--CCCCAACAGTAAAATCGGGTTAATT
GAGTCCTGGGGCAAGTGGTTTGGCCTTTCTGTGCCTCAGTCTCCTCATCTACGAAACGGGGATTAGA
GATACCTTGAACATGGTACTTAATCTCTTAGCATCTCGATCTCTTTATTTT----ATGATGGCAACA
--GATGTTGG-CAGGCCACTTGACCTC-CTGTGCCTCAGTTTCCTCATCTGTAAGAGCGGG------
G--ATGTTGT-CAACCTACATAGATTCTCAATTTTTCTCTTCTGTAAAGTGAGTGACATTTATAATA
GCAAACTTGGGCAAGTTGCTTGACCTTGCTCAATGTCAATATTCTTATCTGTGAAAAGAAGATGATG
GCAACCT---GTGAAC--CTCAACCTCTCTGGGCCTCAGTTTCC-CTCGTGTTAAATGGGCATAATA
GCAACCTTAG----GTTAGTTAACTTCCCTTTGTTTCAGTTTCTTCACCTCTAAAATGGA---AATA
GCAACGTTGGGCGAGTCATTTAAC---
--------------GCAACTTAAA-----TGTGCCTCAGCTTCCTCATCTGTGGAGTGGTGTTTG-G
GCAATTT------GGTCACTAA--CCCTCTGTGACTCAGTG-CCTCAACCGT-----GGGTATCATA
GCACCCTTGGGCAATTTGCTTACCCTTGCTGGACCT--GTCTTTTCATCTGTACGATGGGGATACTA
GCAGGCTTAAGCAAGTTATCTAATCTCCTGGTATCTTAGTTTCTGCACCTATAAAATGGAGGTAATA
GCAGTTCTGGGCGGGT---
--------------GCCACTACTCCTCTCCGGGCCTCAGTTTCCTCCTTTGTTCAAGGAG-------
----------GCCAGTGACTTCACCTCTCTGACCTGCAGTCTCTTCATCAG----------------
--------------------------------GCCTTGGTTTTCCTCTTCTGAAATCGGTGATGATA
GCGACCTTGGGAGGGTTGGGTCCTCTCCCTGGGCCTCAGTTGCCTCCTCTGTGCCCTGGGAGGGTC-
GCGACCTTGGGCAAGTTACTAAAGCGGCTGGGGTCTTGGTGACCTCATCTGTACAATGTCCATGACG
GCGACCTTGGGCAGGTTTCCTAGTCCCTCTGCGCCTCAATTTCCCCACCTATAAAATGGGGA-----
GCGGCCCTGGGAAGGTCACTCG-CCTC--
GGAAACTAAGGCAC-TTGCTTAAAACTTCTG-ATGTGATTTTTCTTAACTATAAAGTAGGGATAACA
G-GAATTCGGGCAA-TCACTTGACCTCTACATGCCTTAGTTTACTTATCAGTAAAATAGGGATAATG
---------GGCAAGCAACTTAACCCCTCAGTGACTCGGTTTCCTCATCTGTAAAACAGGAACAATG
---------GGCAAGTCACT-GGCTTCCCTGGGCCTCAGTTTCCTAATC------------------
GGCACCTTGGACCAATTATGTAAGCATGTTGTGC-TCAGTTTCCTGATCTACAAAATAGGGTTAATA
G--GCCACGAC--AGTTCCCTG-TGCCTCAGAGCCTCATTTTCTTCATCTGCGAGGTGCAGATAGT-
--GGCCTGGTGCCTGTGACCTC-CTCCTCTCGGCCTCAGTTTCCCCGCCCGTAAGACGGGGATGGTA
GGGACCTTCAGCCGGGCCTCTTAGCTCCCTGTGCATC---TTCCTTATCTGGGAAGTGGCAGTTTCA
GGGACCTTGGGCA-----CTTCACCTCTCTG-GCCTCAGTCTCTTCTTCTGT---------------
GGGACTCTGGGTGAATC-CTTTGCCTCTCAGAGCCTGGGTTTCTTGGCTTGTAAAATGGAGATGATA
GGGACTTTAGGCAAATTGTTAATCCTCTCTGATTCTCAATGTCCTCACCTGCAAA--CAGAACAATG
GGGACTTTGAGCAAGCTGCAAAATCTCCCTAAGACATACCGTC-TTACCTTTAAAAGGGAGGACACA
--------GGGCAAGTCACTTCATCTCCCATGTCCTCACCTTTCCTCTCAGTTACATGGTGAG----
--------GGGCAA-TAGCCTCTTCTCCCTGTGCCTCAGCTTCCTCA---GGAAGAGGGGGAGAACA
G-GGCCTCGGGCAAGCTGCTCTGCTTTTCTCTGTCTTAGGGTCTCCGTCTGTAAAATGGG-ATCACA
GGGGCCTCAGGGACAT--CTTTCCCTCTTTTGCTTTTAGTTTCCTCATATGTATAAAGAG-------
GGGGCCTTGGACAAAGCACCTAACGTCTCACTGCTT-GGTTTCCCC-TCTGTAGATAGGAGATGTTG
GGGGCCTTGGGCAACTGACACACCCTCTCTAGGCCTCAGCTAC------------------------
GGGGCCTTGGGCAAGGCACTGTACTGTTCTGGGCTT-GGCTTCCTAGTTTGTACAA--GTGTTAGAA
GGTACAGTAAGCAAGTCACTAAGTCTCCTTAAGTGTAAAATTCTCTATCCA-AAAATGGGTACAATG
GTAACATCAGCCAAGTTATTTCATCTCCCTGTTTC---ATTACCTCATTTGTAAATTGTGGATTATA
GTAACCTTGACCACTAGGCTCAATGGCTGTGT----------TTGCCATCTGTGAAACAAGGATGATA
GTAACCTTGGGCAATTTACGTAATCT-------------GTTTCTTGAACTGTAAACTGGAGATAATA
GTAAC-TCGGCCGAGTTACCTAATCACTCAGTGGTTCAGTGTCCCCATCTGGAAAATGGACTTCTCA
GTAAC-TTGATCAGATTA----AACTCTATTAAC---------------------------------
GTAACTTTGGGCAAGTTACTAAACCTTCTTGTGCGTCTGTTTTCTCATCTGTGAAATGAGCGTAACA
---GTAATAAATA
GTAATCTCGGACAAGTTACCCTACCTCTCAGGACCTCAGCTTCCTTGTCTG----------------
GTAATTTTGG-CAAGTGAGT--ACCTCTCTGAAATTCTGTTTCTTTGTAGGCAAAA-----------
GT-ACATT---CAAATCATCTACCCTATCT------CAGTTTCTTCATCTGTGAAATACAGATAATA
GTAGCCACGGGCAAGCTGCTTTCCCTCTCTAGGCTT--GTTTCTTCCTGCGGAAGCAGAG-------
GTAGCCCTGGGCCTATCACTTAATATTCTTGTGCCACAGTTTC-TTATCTTCAAAATCTGGATAATA
GTAGCCTTAGGCAATCAACGTAACCTCTTTGTGCCTCAGTTTTCTTATCAGACAAATGGGGATAATG
GTAGCCTTAGGCGGGTCGCC-ATCCTCTCTGCG----------------------------------
GTCACCACAGAAAAGTCATCTAACCTCTCGGTGCCCCAGTCTTGTTCTCTGTCCA-TGGCAATTCGG
GTCACCTGGCTTCCTCATCTGAAGCTGGCAGTGTCGCGGCTTCCTCATCTGTCAGAGGGCCACAGTA

GTCACCTTGAGGTGGT--CTTAACCTCTCTGGGACTC--TTTC------------------------
GTCACCTTGGAC-----------------TGTGCCTCAGTCTCCTCTGTCGT------GGGGTGACA
GTCACTTGCCGTGAGTCCTCCAACCACCATGTGGCTTCGTGGCTTCATTTGTAAAGTGGGG-CAAGA
GTCACTTTAATTGAATGGCTTAACTTCTCTGTGTCCCAGTTGAATCATTTGTACAAAGGAGATATTA
GTCAGCTTGCCCCAGTCACCTGCCTTTTGTGT------GTTTCCTCACATATGGAA-----------
GTCATCTTGGACATGCC-TTCAGCCTTTCCGAGTCTGAAGATTTTGCCCTTTGTATTAATTAGAATG
GT--CCTTGGACAAGTGGCTTAACCTTCCTTTGCTTCTGTTTCCTTGCCTACAAAACGGGGATGATC
GT--CCTTGGGCACATTGCACCACCTCTTTGGGTTTCAGTTTCCTTGGCTATAAAGTGGGGATAATA
GT--CCTTGGGCGGGTGACTGATACTCTCAATGAGTCTGTTTTCCCATTTGTAAGATGGGATCCATA
--------------GTCGATTAACCTCTCTACGCTTCATTTTCCCTTGTTTGAAAATGGAGATAGTA
--------------------------------------GTCTCCTCACCTGTGAAATGGGGCTAATG
GTGAACTGAGGCCAGTAGCTCAACTTCTCTGA-----------------------------------
GTGAACTTGTGCAATTTACTT------TCTATACTTCAGAGGGCTGACTTGTAAAGTGGGCTTAATA
GTGACAT--------------AATCTCTTCATGCCTCAGTTCGCTTATTTGTAAAATTAAGTTAATA
GTGACATCCGGCAATTGACAGGTGAGCCCTGGTCCTTAAGGTCTTCCTCTGGAAAATGGGG-TCCTA
GTGACATTGAACAAGTTATT---
GTGACATTGGGGAAGATACTGAATCTTGCTGCAAGTCAGTTTCTGCATTCCTAA-------------
GTGACCC-AGGCAAGTTACTTAACCTCT-TGTGCAGCTGTTT-------------------------
GTGACCCCTGGCAAGCCA----GCCTCCCTGGGTCTCTGTTTCTTCATCAGTAAAACAAAAGGCTTG
GTGACCC-GGGCAGGCTGCTTAACCTCTCTGTGCCCTTGT------ATCTATAAAATGTGG-TAATA
GTGACCC-GGGCA-GTGAGTTCACTTCTCTGTGCCT--GTTTCCTCCTCTATAAAATTGGGTTTTTA
GTGACCCTAATCAGGTCATGTAACATTGCCATGCATCTGCCTCCTGCTCTGTAAAACTGGGATCATA
GTGACCCTAGGCAAGTTACTCAACCTCTCTGGGCCTCATCTCTGACA----CAGGATGACAGCAGTC
GTGACCCTGACCTAGTTCCTTGCCCTCTCTTGATCTCAGTTGCTTTATCTGTAAAAT----------
GTGACCCTGAGCAAATGACTTAAATCCACAGTGACTCAGATTCCTCATCTCTAAAGAATGGGCAC--
GTGACCCTGGACAAACCACTCCCCATCTCTGGGTCTC------------------------------
GTGACCCTGGACAAGTTACCCAGCCTCTCTGGGCTTGGGTGTCCCCTTGGGCAAAATGGG----ATA
GTGACCCTGGGCACGTAACTTGACCTCGCTGTGCCTCAATCCCATCACCTGTAAGACGGTGCACATG
GTGACCCTGTGCTAGTTGCTTAACTTCTCTAAACTTCCGTTTCCCCATCTGTAAAAATAGAA--ATA
GTGACCGCTGGCAAAGTCCTTGACCTCGGCCAGTCTGCGTTTCCTGATCCAGGCAGTGGGGAC----
GTGACCGTGGGCAGGTGACTTGACCTCCCTG------AGTTTCCTCATCTCTAAGATGGGGACAAT-
GTGACCT--
GTGACCTAGAGCAAGTCACCCCATGACTTTGGGTTTCAGTTTCTTCCCCAACAAAATCAGCCAACTC
GTGACCTATAAAAGGTCACTTTATCTTTCCAGGCTTCTGTTTTTTTTTCTATAAAACCAAAACAATA
GTGACCTCAAGCCACTTGCCTGA------------TTGGTTTCCTCACTTACAGAATGAACGTGGGG
GTGACCTCAGACAAATTACTTAACCTCTCTGTGCCTCAGTT--CTTATCTGTGAGATGGAGATAATA
GTGACCTCAGAT--GTTATCTAGATTCTGTATGCTTGGGTTTCTTCATAAGTAACCTGGAGATGGTT
GTGACCTCAGGCAGGTCATCGG----CTCTCAGCTTCAGCTTCTGCACCT-----------------
GTGACCTCAGGCCAGTCAGCACACCTCTCTG------------------------------------
GTGACCTCAGGCCGGTTCTCTGACCTCTCTGTGCCTCCTCTTCCTTCTCTGTAATGTGAA-ACAACA
GTGACCTCAGTCAAGTGGCTTAAACCCTCTGGGCCTAGGTTTTCTCACCTACAAAACGGAGATGACA
GTGACCTCGAG---GTAACGTAACCTGTGTGT-----------------------------------
GTGACCTCGGGAAAGTTAATTAACCTCTCTGTGTTTCAGTCACCTTGTCTTTACAGAGAGGATGTTG
GTGACCTCGGGCAAGTTGCTCGGCCTCTCCGAGCTTCTGTGTCTTCATCTATAGACTGGGCATA-TA
GTGACCTCTAACAAGTTTCTTTATCTCTCTGTGCCT--GTTTCCTCAT------------------A
GTGACCT-------------------CTCTCTACCTCAGTTTTTCCATTTGTAAAATGCGGATAATA
GTGACCTCTGGCAAGCTACT--GTCTTACCTCCTCCAAG---CCTTATCTGTA--ATGGGAATAACT
GTGACCT-GAGCAAGTCATTTCATCTTCTTCTGCCTCACTTTTCCCATATG----------------
GTGACCTGGAGCACATTTCTTAGCCTCTCAGTGTCGCTGTTTACTCATCCGTGAAAAGGAGATAACA
GTGACCTGGATCCAGCTACCCAACC------------------CTCATCTGTGAAGTGAGG------
GTGACCTGGGACAAGTTGCTTTAACCTGATGTGCCACAGCTTCTTTATCTACACAGTGGAGGT----
GTGACCTGGGACAATCCACTCTACCTGTCTATGCCTTGATGAGCTCATCAGTAAAGTGGTGATAGTA
GTGACCT-GGGCAAGTTACTCCATCTCTCTGGGC---------------------------------
GTGACCTGGGCCAAGTTACTTTACTT------GCCTCTGTCTGTTTATTTATAAAATCAAGATAAGG
GTGACCT-GGGCGGGTTACTAAACTTCTTTGTGCCTCAGTTTCCTCATCTGTAAGACGAGGGTAATA
GTGACCTGGGGCAAATCAAC------CTCTGGGCCTCACT---------------------------
GTGACCTGGGGCAGAAGACTT--CCCTTCTGTGCCT-------------------------------
GTGACCTGGGGCATG--ACTTAACCTTT--GCTCTTCAGTC--------------------------

```
GTGACCTGGGTGGAGTTATCAA---TCACGGTGCCCCAGTTGCCTCAGCCATGAAGTGGAG------
GTGACCTTAAATAAATGGCA-GTCCTTTTTTGACCTCAG-----------------------------
GTGACCTTAACTTGGTCAGTTGACATCTCTGGTCTTAAGTTTCCTAATCTGCAACATTTGAAGGTT-
GTGACCTTAAG---ATTCCTTAACCTCTCTGAACCTCAGTTTCCTCATCCATAAAACAAGGAAAATA
GTGACCTTAAGCAAGTCCCGTA-CCTCTCTGGGCCTC--CTTTCTCATCTCTAGAGTGAGGATGATA
GTGACCTTAC-CCAGTGACTCACTCTCTTTGGGCCTCAGGCTACTC-TGTATAAA------------
GTGACCTTA--CCCGTTAC-------CTCTGTGTTTCACTTTTGTCCTCTGTGAAATGGGGATGATA
GTGACCTT-------------ACCCTTTCT------------CATCATATGCAAAATGGCGATAAGA
GTGACCTTAGATACAC-ATCTAACCTCTCTGTCCCTCATTTTTTTCATCTGTAAATTGGGGATACTA
GTGACCTTAG-CAAGACATTTCTCTTCTCTGGAA--CAGTTTCC-----------------------
GTGACCTTAGGCAAGTT-CTTAACTTCTTTGAGCCTCTGTCTTCTCATCCGTAAAATGAGCATAATA
GTGACCTTAGGCGTGTTACTTCAC-TTTCTGAACCTCAGTTTCCTC--TTGTGAAGTGGGGG--ATG
GTGACCTTCAGCT-GTTGCCCAACTGGTCTGAGTCTTGGGTTTCTCAACTGCAAAATGGAGAGGAT-
GTGACCTTCC-TAAGTCACATCCCCTCTCTGAGTCCTGGCTGCCTGATGTGGCAAATGACGGGG---
GTGACCTTG----------------------------------------------------------
GTGACCTTGAACAAGCTA----AGTTGTCTGAGCCATAGTGTCCCCAAGTGCAAAATGGGATAAAAG
GTGACCTTGAATCAACCACTTCATCTTTCTTTGCCTTGGTT-CCTCATCTGCCAAAGAGGGATAATA
GTGACCTTGACCAAATCACGGAATCTCTCC------CAG---CCTCATTCATAAAATGAGGATAATC
GTGACCTTGACCAAGTCCCATCCCCTCCCTGAGCCCCAGTATGTGCATCTGAAAATCGAGG------
GTGACCTTGACCAAGTTACTCAATCTCTTTGTGCCT--GTTTCCTCACTTATACAATAGGTATTCTA
GTGACCTTGAGCAAGTTAGTTAACCTCTCTGTGCCTCAGTTTTCTCATCTGCAAAATGGGGG--GTG
GTGACCTTGAGCAGGTGACTCAGTCTCTGTTTGCCTGA-----------------------------
GTGACCTTGAGCCA-TCACTTAAC-----TCAGCCTCTGCTTTCCTA-CTGTAAAGAGGGGGTAATA
GTGACCTTGAGGAAGTGATTTAACCACACTGTGCCTCAGTCTCATCATCTGAGAAATGGACAT----
GTGACCTTGAGTCATAACCCAAAGCCCTCTGTACCTCCATGTCCCCCTCCATAAACTG---------
GTGACCTTGAGTGGCTG---TGAGCTCTTG--GCCTCGGTTTTCTCATCTGTAAAATGAGA------
GTGACCTTGCACAAGCAATTTGACTTGCTTGTGGTTCAGTGTCATCGTTTGTAAAATGAGGATA---
GTGACCTTG--CAGGTGACTTCATCTAGCTAAGCCT--GTTTCCTCATCTCTAAAATGGAAGTCAA-
GTGACCTTGGACAAATT-CATCATCTGCCAGTGCCTCAGCTTCCTTGACTATGAGATGGGG-CATCA
GTGACCTTGGACAAGTGACTTCACCTCT--GTGCGTCGACTTCCTGCTGTCTGAAATAG--------
GTGACCTTGGACAAGTTATTTAACCTC-------------------ATGTGTCAAGGAGGGAACATA
GTGACCTTGGACATGTTGTGCG-TCTCTCTGCCTCTCGGT-------TCTGTGACCTGGGCATAGT-
GTGACCTTGG-CAATTTTCTT---CTCTTTGAGCCT-AGTTTTCTCATCTGTGTTATAGGAAAAATG
GTGACCTTGGCCAAATAACTC--TCCTTCTGAGCCA-ATTGTCATCATCTATAAGATGAGATTCAT-
GTGACCTTGGCCAGGTGACCCCTCCTTCCTAAGCCTGAACTTTCTCTTCTATACAATCAGCGGG---
GTGACCTTGGGCAAATTACTTATCAT-----------------------------------------
GTGACCTTGGGCAAATTATTTAACCTCTCTATATCTCACTTTCCCCATCTGTAAAATGGGGATAGCA
GTGACCTTGGGCAA----CTTAGCCCTTTTGTGC---AGCTGTAAAATGAGGATGGTGGGCACCCCA
GTGACCTTGGGCAAGGTATT------CCCCTTACCGTATTT------------AAATGGGAATCATA
GTGACCTTGGGCAAGTCACTCAGCCCCTCTGGGCCT-GCTTTCAACATCGATA--------------
GTGACCTTGGGCAAGTCCCTGGTTCATTCTGGGACTCTGTTTCCTC---------------------
GTGACCTTGGGCAAGTTACTTAACCTCTCTGGGCTTCAGCTGCC-CATCTGTAAAATAGGGATAATA
GTGACCTTGGGCAAGTTACTTAACCTTTCTGCGCCTCGCCTTCCCTGTCTCTTAAATGGAGATCATA
GTGACCTTGGGCAAGTTACTTAATGTCTCCATGCCTCAGTTCCTTCACTTTTTCCATGGATGTAATA
GTGACCTTGGGCAAGTTACTTACCCTCTCTGTGCTTCAGTCTTCTCACATGGAAAATGAGACTAATG
GTGACCTTGGGCAAGTTATT--GCCTTACTGGGTCTCAA---------CTGAAAGAT---GATGATA
GTGACCTTGGGCAAGTTGTTTAACTTTGCTATGTCTTAGTATCTTAATCTGCAAAATGGGAATAGCA
GTGACCTTGGGCACAGTCCCTGAATTCTTTGGGCTTCGGTTTTCTTGTCTATAAAATGAGG------
GTGACCTTGGGCACCTTCTTTAACTCCCATGCACC--------------------------------
GTGACCTTGGGCAGGTGACTTCGCCTCTGAGCGCCTCAGTCTCCTCGTCTGAACGATGGGT-TAACA
GTGACCTTGGGCA--------------------------GTTTCTTTCTGTCTAAAATGGG-ACAGTA
GTGACCTTGGGCCA-------------TCTCTAACTAAACTTGTTTATCTTTAAAACAGGTATAATA
GTGACCTTGGGC---CCACCCCATCTTGCTCAGCCTCAG----------------------------
GTGACCTTGGGCGAGCTGACTAATGCCATTGATTCTTGCTTTCCTCATCTGTAAGAGGAA-------
GTGACCTTGGGCTTGTTACT--ACCACTGAG---GTCAGTTTCCTCAAGT---AAACAGGTATAATA
GTGACCTTGGGCTTGTTACTCAGTTTCTCTGTGCCGCAGTTTC-TCATCTGTAAAA-----------
GTGACCTTGGGGAAGTGACATAGCAGCCCTGGGCTT--GTCTCCATGTCTGTA--ATGGACCTAACA
GTGACCTTGGGGAAGTTACTGAACTGTTTTGT-TCTCTGTTTCCTTATCTGTAAAA-GAGGATGTGA
```

```
GTGACCTTGGG-GACTTGATCAAAATCTG---GCCTCTGTTTCTTCATCTGTAAAAGAAGGATAAAC
GTGACCTTGGGGAGGGCACTGAACCTCTCCGAGCCTCAGTTCCCGCCTCTGCAAACTGGGAATAATC
GTGACCTTGGGTAAAC-ATTTAGA------------TCAATTTCCTCAAGCATTTGAAAAGAAAAGGA
GTGACCTTGGGTACATTCCTCAATCTCCCTGATCAGCAATTGC-TCTTCTCTTAAGCAAGCAGTA-G
GTGACCTTGGGTAGGTAGCTTGCCGTTTCTGGGTGTTAGTTTCGCCTTCTGTAAAATAAGGCTGCTG
GTGACCTTGGGTGAGTCACTTAACCTCTCTGTGCCTCAGAGGCCTCATCTATAAAATGAGGGTTTCA
GTGACCTTGGGTGTGTCAC------------------------------------------------
GTGACCTTGGTCAAGCTACTGAACCTCTCTA-ACCTCTAATCCTTCATGTGTAAAATGGAGATAATC
GTGACCTTGGTCAAGTCAATTAACCTCTCTATGTTTTGGTGGTTTTGTGTATAAAATGGGGATAATC
GTGACCTTGTGCATGTCATTTCATCTCTTAGAGTCTCTGTT------TCTGTAAAAGGGAACTAATA
GTGACCTTTAGCAAGTTACTAACCCTCTCTGTGCTTCAGTTTCCCCATC------ACGGAGATAATA
GTGACCTTTAGTAGTATACTTAACTCCTTCATACCTCAGTTT-----TGTGTAAAATAAAAATTATA
GTGACCTTTGCCTGGTCATTT--CCTCCCTGAGCCTCTGTCTCCCCATCTGTGTGGTGAA-------
GTGACCTTTGGCAAGTGAGTCAGACTCTCTGGCCTTCTGT---------------------------
GTGACGATGG-CAAGTCAATTA-----TCTATGA-TCAGTTTCCTTAGCTTTAAAATAGGAATGATG
GTGACTCTGGG-AAGTCACACACACTCTCTGTGCCTCAGGCTCTGGTTCTG-AAGATGGG---AACG
GTGACTCTGGGCAAGTTACTTCATG-CTTTGTGCCTCAGTTTCCTCATCTGTGAAATGGGGATGGTA
GTGACTGAGGGCT-GACATGTCTTCTCTCTGAGCTTCACTCTCC-----------------------
GTGACTGTGGACGTGTGACTTACCCCCTCTGTGCTTCA-TTTCTCCACCTGAGACACAGGACTAACA
GTGACTGTGGGCAAGGTCACCTTCACCTCTCTGTCCCAGCTGCCCCATCTGTGTGATTACACTAATA
GTGACTGTTGGCAACGTGCCTAACATCG-TGTGTCTCAGTGTCACCGTCCGTAAAATGAGGACGCAG
GTGACTTTAGGCAGATTGCACGACCTCTCTGTGTTTCAATCTCCCCATCTATGAAATGAGATTAATA
GTGACTTTGAGTGAGTCACTCCCCATCTCT-------------------------------------
GTGACTTTGGGAAAGTTATCTAACATCTGTATGCCCCATTTTCCTCATCTACAAAATGGAGATAATG
GTGAGCCTGG--------CTTTTCCGTTTTG----TCAGTTGATTCACCTTCAGTCAGG----AATA
GTGAGCTTGGGTGGATTGTCTTA----TCTGATCCTCAGGTTCCTCATTTATAAAACAGAAATAAAT
GTGAGTCACGT--AGTTATTGAATCTGTCTGTGCCTCAATTTCCTCATCTGTAAAATGAGGATAAGA
GTGAGTGTAGGCAAATTACTTAATCTCTCTGAGCCTTCATTTCCTCGACTCCAAAATGGGGATAATA
GTGATCC-AGGCAAGTGACTTCAC------GGACCTCAGTTTCC-CATCTGCAAAATGGG-ATGATA
GTGATCCCGGCCGAGCTATTCTATCTTCCTGTGTCTCAGTTTGCGCATCTGTAAAGTGGGGATAA--
GTGATCCTGGGCCGATGACTCATCTCCTCT--ACCTCTGTTACCTCGTCAGTAAGATAGAGATAACA
GTGATCCTGGGCGAGTTGTTTTACTTCTCTGGGCC-CTGTGACCCCACCTTCAAAATGAG-------
GTGATCT-GGGAAGATGACGTAACC---CTGAGCC-CGATTTTCTTATCGGTGAAATGGG-------
GTGATCTTAGATAAGGATCTTCATCTGTCTCT--TTCCATTTCTTCATCTGAAACCTGGGGCTAATG
GTGATCTTAGGCAAATTACTGAACCTTTCTGTGTTCCAGTTTCTTCAGCTGCAAAGCAGGGACAATG
GTGATCTTA-------TGATCTACCTCTCAGTGCCTCTGTTTACTTATCTG-AAAATGATGACATTA
GTGATCTTCGGCAAGTGACCTAACTTCTCTGATCTTTAGTTTCCCCTCTAGTAAAATGAGGATAATA
GTGATCTTGAGAAAGA-GTTGAGCCTCTCTGGGCCCTGGCTTCCCC---------------------
GTGATCTTGGACAAGTTATTTAACTTTCCTGTGCCTCATTTAACACATATATAACATGGGGATACCA
GTGATCTTGGCCAAGGTGCTTAAATTTCCCAT-CTGTAAAATGAGTATAATGATAATGATGATGACA
GTGATCTTGGGCAAGTAACTTCAGCTTTTTGT--CTCAGTTTCCTCACCTGTAAAATGGGGGTAATA
GTGATCTTGGGTAAACACCTCTGACTCTCTGGGCATCAGTTTCCTCCTTTAGAAAGTG---------
GTGATCTTGGGTAAAT-ATTTAA----TCTGTGCCTCAGATTCCTCACTGATAAAACTGGAATAA-A
GTGATGT-GGACAAGTCACCTGACCCCTCTCGGCCT--GTTTCTTCATCTGTCAAATGGGCGTCATG
GTGATGTTAG----ATTGCATAATCTCTGTGTCC----ACTTTGTCAACTGTAAAATAGAGGGTGGA
GTGATGTTGGACACGTAACTTCAGTTTTCCGAGCTTCAGTTTTATTGTCTGTAAAGCGAGATTAATA
GTGATTCGGGGCATGTCACTTGGCCTCA---------GGTTCCCTCATCTGTACAAT----------
GTGCATCCTGGAA----------------TGTACATTAGTTTCTTCATCTGTTAAAGGGGAATAACA
------------------------------GTGCC-CAGCTCCCTTATCTGTAAAATGGGG-TACTA
GTGCCCTGGAACAAGTGACTTCACCTCTTTGTGCTTGTGT------AAATATGGA---GAGATGATA
GTGCCCTGGGCGGGGAAGGTCACCCTGTGTGCCTCTCGATCCTCTCATCTACACTCAGCTGGTGGTC
GTGCCCTGGGGCAAGGCTTTCACCCTCCTTAGGCTT--GTTTCCTCAGCT-TACAAATGGGCTAATG
GTGCCCTTAGGCCAATTGCTT------TTAGAGCTTCAGTTTTCTCACTTGGAAGATGGGGATAATA
GTGCCCTTGGGCAAATTGCTTACCTTCTCTATCCCACATCTTT------------------ACATCG
GTGCCCTTGGGTAAGTCCCCTAACCCTGCTGTGCCT--GTGTCCCTCACTACATCACAGGTG-AGTG
GTGCCTTCACAAAAGACATGTGACCTCTCTGCGCCTTAGTCTCCTCATCTGAAACATGGGAACA---
GTG-CCTTGGGTCGGATACTTAACCTCTCTGAGTCTTT-TTTCCTCCCCTGTAAAATGGGGGTAATA
GTGCCTTTGGTTAAGTTCCTGAACTGCTCTAAGCCTTAGTTTTCCCATATGTAAAATGGGCTTAATA
```

```
GTGCTCTTGTATACGCCAGT-ATGCCCTCTAGTCTTCATTATCCTCATCTGTACAGTAGAGATAATA
GTGCT----GGTGGGTTATTA---CCCTCATTGCCTCAGTTTCCCC-TCTGCACAATGGGGCTGGCG
GTGGCCACAG-TAAGCCACTC-----CTCTGTGCCTCAGCTTT-TCTCTAGCAAAATAGA-ATGCTG
GTGGCCCTTGGTG-------------CTCTGTGCCTCAGTTTCCTTCTGT-TAAACTGCAGTTAATA
GTGGCCGTGGGCAAGTTACTTCATCT-TCTGTGCCTTTGTTTCCTGGCATGCATAAGAAGAATCATC
GTGGCCTGGGGCAAATTAC-------------GCCCTGGGACTCTCACCCGCAAACCAGAGATAATA
GTGGCCTTGAAAAAGCTACTTAAACTTTCTAAACTTGAGCTTACTCATATACCAAATGGAGATGACA
GTGGCCTTGAGCAA----CTTAATCTCTCTGTGCCTCGGGTTTCCTATGTGTAA---------GATA
GTGGCCTTGGGCAAACT-TCTAACCTTGCTGT----CTGTTTTCTCATCTGTAAAATGAGGATAATA
GTGGCCTTGGGCAAATTGTTTCACCTCTCTGTGCCCCAGGATCCTTATCTGTAAAA-----------
GTGGCCTTGGGCAAGACATTTTACCTA-CTGGGCCTCAGTTTCCTCATTTGTAAAACAGGGCAACTA
GTGGCCTTGGGCAAGTCACTCA-CGCCTCTGTACCTGAGCATGCACATTGGCAAAATGGGAACCGTA
GTGGCCTTGGGCAAGTCACTTAACTTCTCTGTGATGCAGCTTTCTCACCTGTGCTGTGGGGACAGTC
GTGGCCTTGGGCAAGTCAGTCCATCTTCCTAGGCCTTGATTTCC-----------------------
GTGGCCTTGGGTAAGTTGCATAAGCT-TCTGT-----------------------------------
GTGGCTTGGGGCAGATTACTTAACCTCTTTAGGTTTCGGTTACTTTACCCATAAAGTGCAGATAATC
GTGGCTTTCTATAAGTCACTTTACTTTACTGTGCCTACGTTTCCTAATTTGTAACATGAGGAGAATA
GTGGCTTTGAACAAGCCACTTGACCACTCTGTGCTCCGGTTTCCCAGCTGGCAATTAGAGGATGGG-
GTGGCTTTGGGCAAGTTACTTAATCTCTCTGTGCTCCAGTTCCCTCTTGTGCCCCAAAGGGATAATT
GTGGTATTGGGTTGGTTACCTAAACTGTCTATGCTCCAGTTTCCTGATTTACGAATTTGGGATAA-A
GTGGTTCTTAAGAATTCATCT------TGTGGACCTCAGTTTCCTAATTTATGAAATGAAAGGGTTG
GTGGTTTTGGA-AGCTTGCCTAACCACTCTATAAACCAGTGTCCTGACCTGTA-----GGAACAC--
GTGTC--------------------------------------------------------------
GTGTCCATGGGCATGTTACTTCATCTCTCTGTGCATCAGATTTCCCATCTCTAATATGGGGACAATG
GTGTCCCTGGGCAAG-----------CTCCGTGTCCCTGTGTCCCTG-CTATAAAATGGG---AATG
GTGTCCTCGGACATATGACTTTCCCTCTCCATGCCTCAGTTTCCCCATC------------------
GTGTCCTCGGGCAAGTATCCTT-CCTTTCTGAGCCTCTGGGCTTTCTTTGGGAAAATGCGGGGCTGG
GTGTCCTCGGGGAGCCAGCCTTCTCTCTCTTGGCCTTAGCTTGCTCCTCTGTCCAGTGGGG--GGTG
GTGTCCTTGGGCAAGTTC----ACCTCTCTGAACTT-GGTT--------------------------
GTGTCCTTGTGTGAGTCACATCACCTCTCCAGACATCTGTCTTGTCATCTGCAAAATGGGGAGGAGG
GTG-TCTCAAACAAGTCACTCAGTCTTGCCTGCCGTCAATTTTCTCACCTGTAAAATAGTAGGAG--
GTTAACACGAGCAAGTGATTTAACCTCTTGGTTCCACATTTCACCTA-CTGTCATTTGGGGATTGTT
GTTAACCTTTGAGTTTCACTTAACTTCTCTGTGCCTTAGATTTCG-ATCTGTAAAATGGGGGTGACA
--------------GTTAATAAACCTTTCAAAATATCAGTTTC-TCGTCTGTAAAATG---ATGACA
GTTACCCTGGACAAGTTATTTGCCTTCTCTGTGCTTTAGTGTCCCCATCTCAAAGTAGGGATAATA
GTTACCTTGAGCAAGT-ATTTATTCTGTCTATTTCTGATTTCCTTTG--TGATGAACAG---CATTA
-----------------------------TAAACTTTCTGTTCGTCATCTAGAATAGGGAGATAATG
-TAACCTTGGG-AAGACACTTA---TCTCTCTGCCTCAGTTTCTCCGTTTGTACAACGAGAA-ATTA
----------------TACTCAACTTCCTCCTGCCTCGATTTCCTCATCAGTAAAATTGAGGGAATT
----------------TACTCCATCCCTTTGAACCTCAGGTTCCTTGTTTGTGAGACTTTGG-----
TCAGCCTGGAGCAAGTC-TCCGACCTCAGCATTCCTCGGTCTCCTCG-------AGTGGGGCCG-TA
TCCACCTTAGACAAATAACTCACTCCCCC--------------------------------------
------TCGGACAAGTCGCTTCACCTCTCAGAGCCTCAAAGTCCTCAT-------------------
---------------------------------TCTCAAGTTCCTCATCTGCAGAATGGAAATAAGA
-----------------------TCTGAGCCTTATTTAGTGTTCTCATCTGTAAAACGGGGAAATTG
-------------------------------TGAATCCACATCTTTATCTGCAAAATCAGGAG----
-TGACCTAGGACACATTTCTCACGCTCTCTG------AATTTC---ATCCATAAAACGGGCAAAACA
-TGACCTTAAGCTAAT-----AACCTCTTCATGACTCTATTTCCCTGCTTGTTAAATGGGACC--CA
-TGACCTTCAACAAATCATTTCACCTTTGTCAGCCTAGCTTTTCTC-TGTGTAGAATGAGGGG----
-TGACCTTGAACAAGCTCCTCCATCGCTCCAGGCCTCGTTTTCAGGTGTATATGATG---------
-TGACCTTGAGCAAATTACCCAACCTGT-------------TCCTTATTTGTT-------------
-TGACCTTGGCCAGGC-ACTCA--CATCCTGTGCCT--GTTTTCCCGTTTGTCAAGTGG--ACAGCC
-TGACTTCAAGAGAGTTTCCTTAGTTCTGTAAGGAAAAGCT-CTTCATCTGTTAAATGGGGGTGACA
-TGACTTCAGGAAAACCACTCGACTGCACTGAGCTTCAACATCTTCAGCTGCAAGGCAGGAA-AATA
-TGATCTTGGACAACTGATCTCAAATCTCAATGACTTTGTTTCTTCATATGTGATATGGAGATAGAG
--------------------------------------------------------------TGATT
-TGCCATTGGCTGTGTTACTT----TTCTGGGGTCTCAGCTTCCCCTTCTGTAAAGCAGGGAGAATA
-TGCCCTGGGGCAAGGTACTATCCTATTTTGTGCCT-GGTCTCTTCATTTGAATAATAG--ATAATA
```

```
TGGACCTTGAG--------------------------------------------------------
-----------------------------TGGACTTCA-CTTCATCATCTCTAAAATGAGATTAAAG
-TGGCCATGGACAAGTTTCATACCTTCTTTGAGCCTTGGCT-CCTCATCTGTTAGATGTGAATGGGA
-TGGCCCTGGGCAAATTACTTAATCCCTCTAAGGCT--ATAGCCTTATCTCTGCA-TGAGGATAAAA
-------------------------------------------------TGTAAAATGGGGAGAATG
-----------------------------TGTGCCTCAGTGTCCCCAGCTGTGACATGGGTACAGGA
---------------TTACTCAACTTCTTTCTTTATCGATTTCCTCATCTATAACATGTCAACAATG
-TTACTTTGGGCAAATGGTTTAACCTGTCTGTGCTGTGGTTCCCTTGT-TAAAAAATGAGGATAGCA
-------------------TTCACCTGTCGCTACCTCAGTTTACCCATCTGCAAGATGCAG-TGGT-
------TTCAGCAGAGTTCTTTCTGTCACTTTGTGCTCGTTTCCGCATCTGTAGAATGGGGGTAACT
TTGACAT--GGCAAATT-----ACCTCTCTGC-CCTTAGTTTCCTCATTTGTAAAATGGTAATAATA
TTGACCTT----AAGCCTCCTGACCTGTCTGGGCCTC---CTCCTCCTCAGTAAG------------
TTGACCTTGGGCTAGTTACTTAACCATTCAGTGCCTCAGTTTCCTCATCTGTAAAGAGGAGATAATA
-------------------------TTTCTAAAACACAGTTTCCTTGTCTGTAAAAGGAGGG-----
```
